# Using Data from Macaques To Predict Gamma Interferon Responses after Mycobacterium bovis BCG Vaccination in Humans: a Proof-of-Concept Study of Immunostimulation/Immunodynamic Modeling Methods

**DOI:** 10.1128/CVI.00525-16

**Published:** 2017-03-06

**Authors:** Sophie J. Rhodes, Charlotte Sarfas, Gwenan M. Knight, Andrew White, Ansar A. Pathan, Helen McShane, Thomas G. Evans, Helen Fletcher, Sally Sharpe, Richard G. White

**Affiliations:** a TB Modelling Group, CMMID, TB Centre, London School of Hygiene and Tropical Medicine, London, United Kingdom; b Public Health England, Porton Down, United Kingdom; c Imperial College, London, United Kingdom; d College of Health and Life Sciences, Department of Life Sciences, Brunel University, London, United Kingdom; e The Jenner Institute, University of Oxford, Oxford, United Kingdom; f TomegaVax, Portland, Oregon, USA; g Immunology and Infection Department, London School of Hygiene and Tropical Medicine, London, United Kingdom; IIS/LAD/NIAID/NIH

**Keywords:** nonhuman primates, T-cell immunity, bacillus Calmette-Guérin, interferons, mathematical modeling, tuberculosis, tuberculosis vaccines

## Abstract

Macaques play a central role in the development of human tuberculosis (TB) vaccines. Immune and challenge responses differ across macaque and human subpopulations. We used novel immunostimulation/immunodynamic modeling methods in a proof-of-concept study to determine which macaque subpopulations best predicted immune responses in different human subpopulations. Data on gamma interferon (IFN-γ)-secreting CD4^+^ T cells over time after recent Mycobacterium bovis BCG vaccination were available for 55 humans and 81 macaques. Human population covariates were baseline BCG vaccination status, time since BCG vaccination, gender, and the monocyte/lymphocyte cell count ratio. The macaque population covariate was the colony of origin. A two-compartment mathematical model describing the dynamics of the IFN-γ T cell response after BCG vaccination was calibrated to these data using nonlinear mixed-effects methods. The model was calibrated to macaque and human data separately. The association between subpopulations and the BCG immune response in each species was assessed. The macaque subpopulations that best predicted immune responses in different human subpopulations were identified using Bayesian information criteria. We found that the macaque colony and the human baseline BCG status were significantly (*P* < 0.05) associated with the BCG-induced immune response. For humans who were BCG naïve at baseline, Indonesian cynomolgus macaques and Indian rhesus macaques best predicted the immune response. For humans who had already been BCG vaccinated at baseline, Mauritian cynomolgus macaques best predicted the immune response. This work suggests that the immune responses of different human populations may be best modeled by different macaque colonies, and it demonstrates the potential utility of immunostimulation/immunodynamic modeling to accelerate TB vaccine development.

## INTRODUCTION

Tuberculosis (TB) disease remains a major global health problem (1), and Mycobacterium tuberculosis bacillus Calmette-Guérin (BCG), the only licensed TB vaccine, exhibits variable efficacy (2, 3). In order to reach WHO TB control goals, a new, effective vaccine is vital (4). Animal models are used in almost every aspect of vaccine development, including helping to understand the transmission dynamics of the disease and the immunogenicity and efficacy of vaccines (5). They are therefore a vital and efficient tool in vaccine development (6). In preclinical TB vaccine research, nonhuman primates (NHPs) are a valuable animal model (7, 8) and are genetically and physiologically more similar to humans than small animals with respect to TB disease and immune response (7, 9).

Historically, rhesus (Macaca mulatta) (10) and cynomolgus (Macaca fascicularis) (11) macaque species have been used as the primary NHP models in TB vaccine research (12–14). Both species have been shown to respond to BCG vaccination, which affords them partial protection from TB (15–19); however, it has been shown that the same experimental conditions (infection with Mycobacterium tuberculosis following vaccination or a vaccine immune response) may lead to divergent outcomes for the two species (7, 20–22). Furthermore, the colony (country of origin) of macaque, even within the same species, has been shown to affect the level of protection against infection and the level of response after vaccination. For example, differing levels of protection have been observed for Chinese and Mauritian cynomolgus macaques: Mauritian cynomolgus macaques developed end-stage progressive TB in 7 weeks, while Chinese cynomolgus macaques remained healthy past the end of the study (12 weeks) (23).

These differences suggest that the immune responses of different human populations (e.g., those with previous BCG vaccination or those who are BCG naïve) may be best modeled by different macaque colonies. In 2014, the Bill and Melinda Gates Foundation adopted a new strategy for the selection of new TB vaccine candidates for clinical testing based on immune response and challenge results in NHPs (24). Therefore, in order to increase the likelihood of developing an effective vaccine, it is critical to identify and understand differences between macaque populations.

Here we focus on establishing the most representative NHP model for modeling the gamma interferon (IFN-γ) immune responses of adult humans in the UK following recent BCG vaccination, as one example of the prediction of vaccine immune responses in humans from a macaque animal model.

For this purpose, we conduct a proof-of-concept study to evaluate the potential use of novel immunostimulation/immunodynamic (IS/ID) modeling methods in vaccine immune response translation between species. A mechanistic mathematically based approach is used to quantify the dynamics of the immune response. By building the mathematical models on the basis of quantitative immunological data, it is possible to describe how these mechanisms may differ within and between species and to draw quantitative comparisons. Such modeling techniques are commonly used in drug development (pharmacokinetic/pharmacodynamic modeling) to translate drug responses between species (25–27) but have yet to be used in vaccine development.

First, we develop a model of IFN-γ-producing CD4^+^ T cell dynamics after BCG vaccination and assess the suitability of the model structure for predicting responses by calibrating the model to the data (analysis 1). We investigate the impact of the human and macaque population covariates to explain the within-population variation in responses, which our previous analysis on humans (28) showed can have a substantial impact on the magnitude of the response (analysis 2). We then test which calibrated macaque models best predict human IFN-γ responses (analysis 3). Finally, we use the calibrated mathematical models for macaque and human subpopulations to predict the dynamics of the constituent T cell populations over time (analysis 4).

## RESULTS

### Analysis 1. Calibration of the model to IFN-γ data and exploration of model predictions for macaques and humans separately.

**FIG 1:**
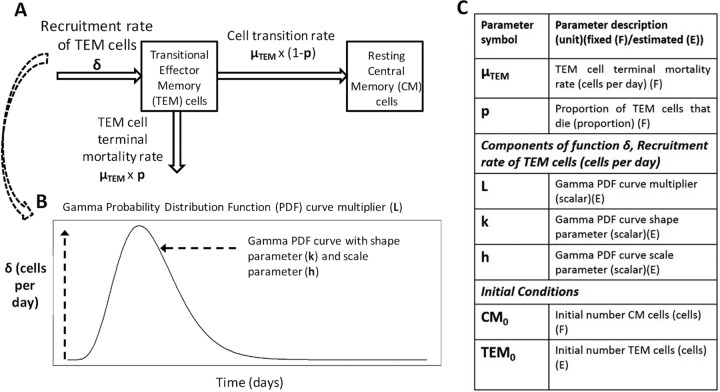
(A) Schematic of the mathematical model representing the immune response dynamics of two CD4^+^ T cell populations secreting IFN-γ. (B) Depiction of the changes in the recruitment rate of transitional effector memory cells (δ) over time. (C) Key model parameters. Equations may be found in the supplemental material.

**FIG 2:**
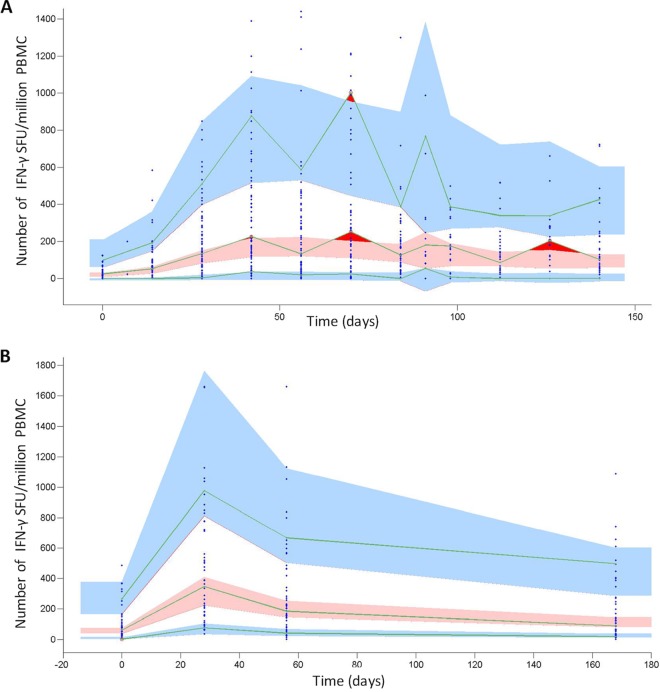
VPC plots showing the number of IFN-γ SFU per million PMBC, by time (days) for all macaques (A) and all humans (B). The VPC plot assesses the appropriateness of the proposed mathematical model (Fig. 1) for describing the empirical data by comparing the data simulated using the model, the population mean parameters, and associated variances (Table 1) to the empirical data distribution (see the supplemental material for more details). Blue points show empirical data. Pink regions represent the ranges of the medians of the simulated data for 500 simulations. Blue regions represent the ranges of the 90th and 10th percentiles of the simulated population data. The green lines link the empirical percentiles (10th, 50th, and 90th). Dark red regions show where the empirical data fall outside the ranges of the simulated percentiles. The lack of dark red regions (aside from cases in which data are variable between time points in macaques) indicates that our proposed mathematical model (Fig. 1) adequately represents the empirical data.

Our mathematical model representing the immune response dynamics of two CD4^+^ T cell populations secreting IFN-γ is diagramed in Fig. 1. The estimated parameter values for both macaques and humans can be found in Table 1. The visual predictive check (VPC) plots in Fig. 2 show that the ranges for macaques and humans in the model simulation cover the empirical data, indicating that our model yields a good representation of the empirical data. Further diagnostic plots and model prediction plots can be found in Fig. S3 to S7 in the supplemental material.

**TABLE 1:** Population mean parameter estimates for analyses 1 and 2 for macaques and humans^*a*^

Parameter or statistic	Macaques					Humans				
	All (analysis 1)		Covariates (analysis 2)			All (analysis 1)		Covariates (analysis 2)		
	Value	RSE (%)	Subpop.	Value	RSE (%)	Value	RSE (%)	Subpop.	Value	RSE (%)
Parameter (unit)										
Initial no. of TEM cells (TEM_0_) (cells) (E)	20.7	29	Chi	0.29	39*	59.9	17	BCG: Y	149	15
			Maur	65.1	24					
			Indo	23.2	41*			BCG: N	30.6	14
			R: Ind	15.7	20					
Gamma PDF curve multiplier (L) (scalar) (E)	1,170	13	Chi	617	43*	1,490	14	BCG: Y	3,240	14
			Maur	1,460	28					
			Indo	1,100	45*			BCG: N	747	14
			R: Ind	1,250	14					
Gamma PDF curve shape parameter (k) (scalar) (E)	3.31	5	Chi	4.3	11	1.45	9		1.55	16
			Maur	3.15	10					
			Indo	3	20					
			R: Ind	3.53	6					
Gamma PDF curve scale parameter (h) (scalar) (E)	15	8		13.8	7	18.4	18	BCG: Y	21.7	24
								BCG: N	15.2	34*
Initial no. of CM cells (CM_0_) (cells) (F)	0			0		0			0	
TEM cell terminal mortality rate (μ_TEM_) (/day) (F)	0.1			0.1		0.083			0.083	
Proportion of TEM cells that die (p) (proportion) (F)	0.925			0.925		0.925			0.925	
Within-population variation (WPV) (%)										
Initial TEM cell population (TEM_0_)	130	25		41	27	107	15		52	19
Gamma PDF curve multiplier (L)	96	13		90	13	95	10		61	12
Gamma PDF curve shape parameter (k)	24	24		23	24	25	28		32	33*
Gamma PDF curve scale parameter (h)	19	21		21	20	58	25		43	37*
Goodness-of-fit statistics										
−2LL	7,209			7,183		2,738			2,653	
BIC	7,253			7,251		2,779			2,706	

a For details on the parameter-covariate relationship, see the supplemental material. F, fixed; E, estimate; TEM, transitional effector memory; CM, central memory; PDF, probability density function; RSE, relative standard error; subpop., subpopulation; Chi, Chinese cynomolgus macaques; Maur, Mauritian cynomolgus macaques; Indo, Indonesian cynomolgus macaques; R: Ind, Indian rhesus macaques; BCG: Y, human participants who were BCG vaccinated at baseline; BCG: N, human participants who were BCG naive at baseline; −2LL, −2 log likelihood; BIC, Bayesian information criteria. RSEs of ≥30% are marked with asterisks.

### Analysis 2. Population covariate impact on within-population variation in model parameter estimates.

**FIG 3:**
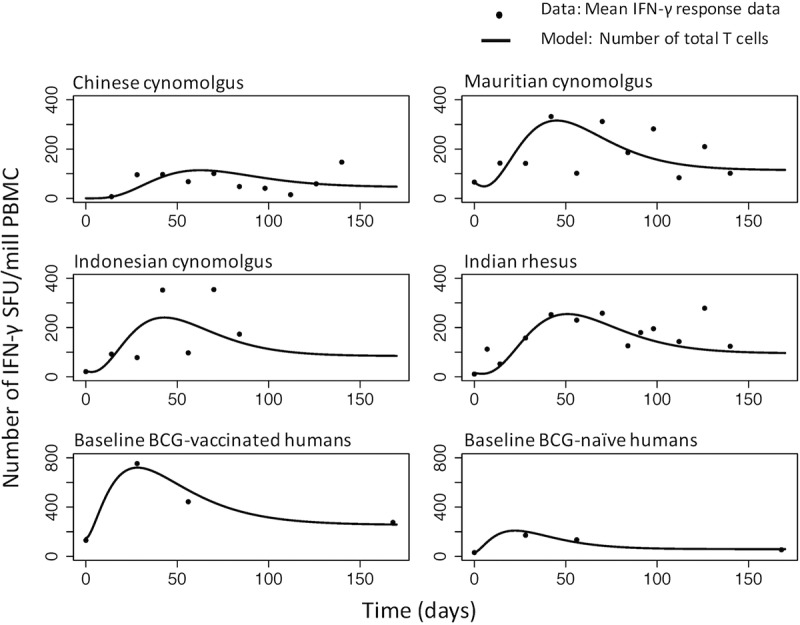
Total number of T cells secreting IFN-γ (the sum of the number of transitional effector memory cells and resting central memory cells) over time. Each point represents the mean of the data at a particular time point. Lines represent model predictions. Model predictions use the estimated subpopulation model parameters from Table 1 for the four macaque colonies and the two human subpopulations with different BCG statuses. (Note the differences in scale between macaques and humans.)

We found two covariates to be important: stratifying macaques by colony and humans by baseline BCG status reduced the within-population variation in the initial transitional effector memory cell count (TEM_0_) for macaques, TEM_0_ for humans, and the human gamma probability density function (PDF) multiplier and scale parameters (parameters L and h) (Table 1; see also Tables S7 to S13 and Fig. S8 to S12 in the supplemental material). The VPC and further diagnostic plots for the subpopulation models show that the model describes the data adequately (see Fig. S13 to S18 in the supplemental material). Accounting for the population covariates reduced the Bayesian information criterion (BIC) value significantly, by 73, for humans from that in analysis 1 (BIC values, 2,779 in analysis 1 and 2,706 in analysis 2 [Table 1]) and decreased it by 2 for macaques (BIC values, 7,253 in analysis 1 and 7,251 in analysis 2 [Table 1]). The model-predicted total mean number of IFN-γ-secreting cells (transitional effector memory [TEM] cells plus central memory [CM] cells) over time is shown in Fig. 3 as a visual assessment of the goodness of fit of the model to the mean empirical data. Also, Fig. S19 and S20 in the supplemental material show the 10th to 90th percentiles of model predictions after accounting for within-population variation.

### Analysis 3. Which macaque subpopulations best predicted immune responses in different human subpopulations?

**FIG 4:**
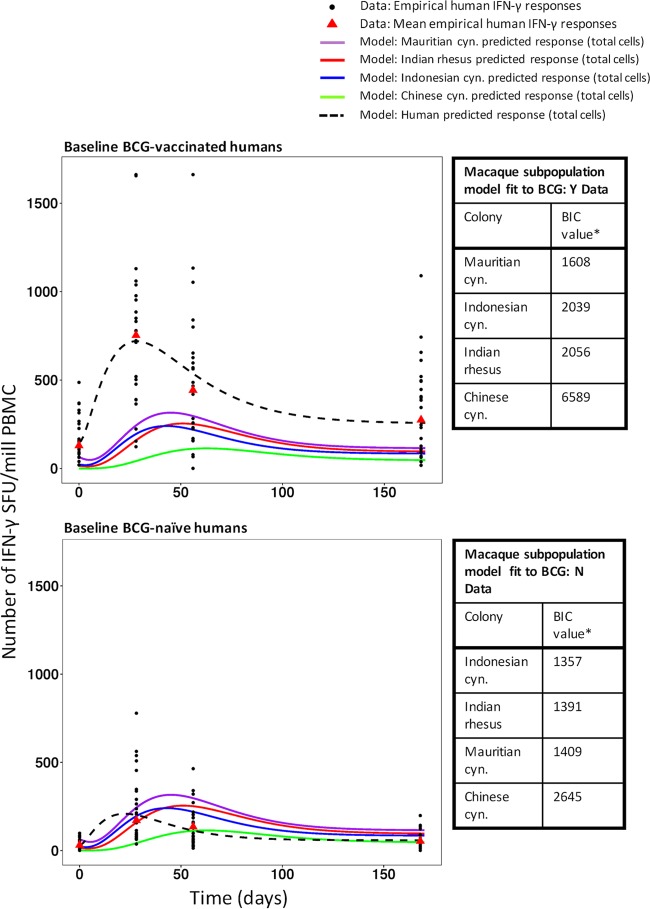
Mean immune responses of the four macaque colonies and of human subpopulations that were BCG vaccinated (BCG: Y) or BCG naïve (BCG: N) at baseline. Empirical data for human responses are represented by black points (individual data) and red triangles (means). Lines show model predictions. The tables show the results of assessments of the ability of the calibrated macaque colony mathematical model parameters (Table 1, analysis 2) to describe the data for the human BCG: Y and BCG: N subpopulations. Bayesian information criterion (BIC) values are listed in ranked order, from lowest to highest. Asterisks indicate that all differences in BIC values are significant (a BIC value difference of >6 is considered significant [46]). cyn., cynomolgus.

The calibrated model for Indonesian cynomolgus macaques from analysis 2 provided the lowest BIC values for the human population that was BCG naïve at baseline (BCG: N), and that for Indian rhesus macaques provided the second lowest BIC value (1,357 and 1,391, respectively [Fig. 4; see also Fig. S20 to S27 in the supplemental material]). The calibrated model for Mauritian cynomolgus macaques best represented the human population that had already been BCG vaccinated prior to baseline (BCG: Y) (BIC value, 1,608 [Fig. 4; also Fig. S21 to S28]).

### Analysis 4. Predicted numbers of TEM and resting CM cells over time.

**FIG 5:**
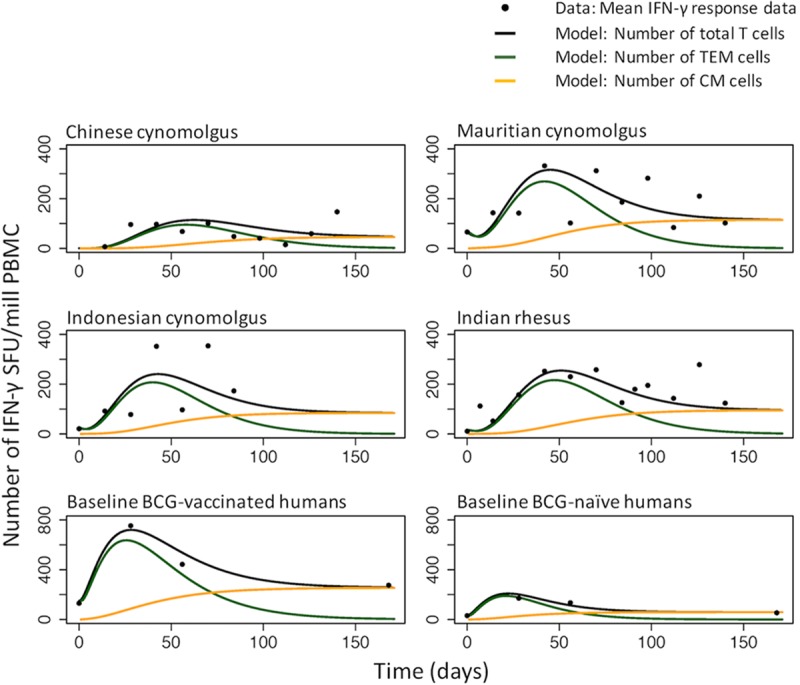
Mean IFN-γ response data (black points) and model predictions for the total number of T cells secreting IFN-γ (black lines), the number of transitional effector memory (TEM) cells (green lines), and the number of resting central memory (CM) cells (orange lines) over time. Model predictions use the estimated subpopulation model parameters from Table 1 for the four macaque colonies and the two human subpopulations with different BCG statuses. (Note the differences in scale between macaques and humans.)

Figure 5 shows the model-predicted numbers of total (transitional effector memory and central memory) cells secreting IFN-γ, over time, for the mean macaque and human subpopulation data. These model dynamics present a prediction for the phenotypic behavior of CD4^+^ T cells and the ways in which they differ between species and subpopulations, which can be validated experimentally.

## DISCUSSION

In our proof-of-concept study, we applied novel immunostimulation/immunodynamic (IS/ID) modeling to BCG immune response data and found that the macaque colony and the human baseline BCG status were significantly (*P* < 0.05) associated with the BCG-induced IFN-γ immune response. No other population covariates were significantly associated. For baseline BCG-naïve humans, Indonesian cynomolgus macaques and Indian rhesus macaques best predicted the immune response. For baseline BCG-vaccinated humans, Mauritian cynomolgus macaques best predicted the immune response.

A key strength of this proof-of-concept study was the application of mathematical modeling techniques to vaccine data that are rarely explored quantitatively. We used established robust quantitative and statistical frameworks (compartmental mathematical models with nonlinear mixed-effects modeling [NLMEM] [29]) to explore the complex biological dynamics, giving an early example of the utility of IS/ID modeling. The biological data we used were standardized between species, with respect to time points and laboratory techniques, which allowed a direct comparison of the immune responses to BCG vaccination.

Although our model was a highly simplified version of the complexities of the immune system (see the discussion in the supplemental material for the main assumptions and their impact [Table S14]), analysis 1 showed that the model described the data well. The model was also a good description of the subpopulation data in analysis 2. However, when the model was calibrated to smaller subpopulation sizes (especially for the Chinese and Indonesian cynomolgus macaques), the estimated model parameters were more uncertain than for the larger populations (see the relative standard error [RSE] values in Table 1). Access to larger data sets on these populations would increase the certainty of the parameter estimates. Additionally, in analysis 2, our aim was to establish how population covariates affect the model parameters using a stepwise addition method. However, as Whittingham et al. point out, there are inherent drawbacks with such a method, despite its widespread use (30).

By modeling the recruitment rate of transitional effector memory cells by the function δ, we were able to represent the nonlinear stimulation of the CD4 T cell response following BCG vaccination, allowing comparison of the dynamics of the response between subpopulations. However, since the recruitment rate of transitional effector memory cells was not based on biological data and was characterized by a theoretical shape, it is difficult to make direct biological interpretations of the parameters. To incorporate a mechanistic stimulation curve in future work, data on the cells involved in the stimulation response would be required.

The results in this analysis were consistent with previous work, in which we applied descriptive statistics to the human data (28). In that study, men experienced a higher baseline IFN-γ response (*P* < 0.1) than women. A similar pattern can be seen in the current work: the median initial number of transitional effector memory cells (TEM_0_) for men is higher than that of women (Fig. S8 in the supplemental material). Additionally, the model in analysis 2 is consistent with our previous findings (28) for humans, in which immune responses were higher in magnitude and were sustained longer for baseline BCG-vaccinated humans than for baseline BCG-naïve humans. Therefore, our results suggest that BCG revaccination provides a higher and more sustained IFN-γ response than primary vaccination in humans. Finally, our results suggest that there are differences in BCG response between different colonies of macaques. This is consistent with work by Langermans et al., who show that rhesus macaques experience a higher IFN-γ response 13 weeks after BCG vaccination than cynomolgus macaques (22), although the potential effect of the colony on IFN-γ response was not highlighted in that work. Differences in responses across macaque colonies have also been found in M. tuberculosis challenge studies: Sharpe et al. showed that the AUC_12Week_ (area under the concentration-time curve at 12 weeks) values for IFN-γ-secreting CD4 T cells were significantly higher for Indian rhesus macaques than for Indonesian cynomolgus (21). Although we do not consider M. tuberculosis challenge in our analysis, these differences may be important to consider when one is selecting an NHP model for human mycobacterial immune response.

Our results imply that responses in Indonesian cynomolgus macaques, followed by those in Indian rhesus macaques, most closely resembled the response in primary-vaccinated humans determined by enzyme-linked immunospot (ELISPOT) assays. However, we approach this conclusion with caution, since the sample sizes of the macaque colony subpopulations were variable. With these smaller sample sizes, model parameterization and validation are less reliable than for larger groups. More data on the colonies with small sample sizes should be collected and remodeled to verify our results. Nevertheless, the large sample size obtained for the Indian rhesus macaques was collated over decades of experimentation. Conventional vaccine studies in macaques are often limited to 6 to 9 animals per group due to space and cost. These smaller macaque experiments are then used to inform clinical vaccine trials, making our small sample sizes more representative of current vaccine development than the large rhesus macaque data set.

It should be noted that in terms of BCG vaccination history, the baseline BCG-naive human subpopulation is the most comparable to all of the macaque subpopulations. Mauritian cynomolgus macaques mounted the highest response to a primary BCG vaccination, and therefore, their data most closely resemble those for revaccination in humans. However, it is apparent from Fig. 4 that the BCG-vaccinated humans experienced a considerably higher magnitude of responses than all of the macaque subpopulations (which were BCG naïve at baseline). This suggests that the immune response to an antigen encountered for the first time is lower and slower than the response induced to the same antigen on subsequent occasions (31). Our results therefore suggest that a revaccinated macaque animal model may be most appropriate for revaccinated humans. This should be considered in further IS/ID translational analysis between macaques and humans.

In our analyses, we consider only a UK-based human population. In future evaluations, an analysis similar to that presented here could be carried out on populations from various geographical locations, since BCG responses have been shown to differ by geographic location (32). Other population covariates, such as age, may also be important (8). Additionally, the question of whether this analysis will be appropriate for other candidate vaccines would benefit from further scrutiny.

Figure 5 explores the dynamics of the constituent T cell populations and provides insights into how and when memory may be developed—an important consideration in vaccine regimen design, i.e., the timing of revaccination and differences between subpopulations. However, we do not currently have data to support these dynamics, so future work could be undertaken using flow cytometry to characterize the relative numbers of complex phenotypic cell types over time and thus to inform models that can provide a better understanding of T-cell dynamics.

In this analysis, we used solely IFN-γ as a proxy for BCG vaccine immunogenicity (33) and did not consider BCG efficacy measures explicitly. We understand that in order to develop a vaccine, both immunogenicity and efficacy are vital considerations. Therefore, in predicting which macaque model best represents the human vaccine response, vaccine efficacy cannot be ignored. However, to incorporate efficacy would require more-complex models and data than those we present here. As more immunological information or functional parameters become available, IS/ID modeling methods will allow us to easily integrate new information, e.g., on cytokines, cells, or (for efficacy measures) bacterial counts. Thus, we will be able to make decisions on the best NHP model to use based on a more complete vaccine performance framework.

### Conclusion.

This work suggests that the immune responses of different human subpopulations may be best modeled by different macaque colonies, and it demonstrates the potential utility of immunostimulation/immunodynamic modeling to accelerate the development of TB vaccines.

## MATERIALS AND METHODS

### Data.

Data on the number of purified protein derivative (PPD)-stimulated CD4^+^ T cells secreting IFN-γ (in spot forming units [SFU] per 1 million peripheral blood mononuclear cells [PBMC]), measured by an *ex vivo* IFN-γ ELISPOT assay, were available for 55 humans and 81 macaques. BCG vaccination was given on day zero, and ELISPOT measurements were performed up to 140 days after vaccination. The details of the human data set and laboratory techniques have been published previously (28). Briefly, healthy UK volunteers aged 18 to 55 years, either with no history of BCG vaccination or previously immunized with BCG, were given 100 μl of BCG, administered intradermally in the upper arm. Immune responses to BCG were measured using an IFN-γ ELISPOT assay at weeks 1, 4, 8, and 24. For demographics and laboratory details, see the supplemental material (Table S1; Fig. S1). All macaque studies were conducted in accordance with the Home Office (UK) Code of Practice for the Housing and Care of Animals Used in Scientific Procedures (1989) and the Guidelines on Primate Accommodation, Care and Use of the National Committee for Refinement, Reduction and Replacement (NC3Rs), issued in August 2006. All animal procedures were approved by the Public Health England, Porton Down Ethical Review Committee, and were authorized under an appropriate UK Home Office project license. Vaccination, sample collection procedures, and immunological methods are described in full in references 19, 23, 34, and 35). All macaques were demonstrated to be mycobacterially naïve prior to BCG vaccination and were between the ages of 3 and 14 years. The human population covariates were baseline (before vaccination at time zero) BCG vaccination status (either BCG vaccinated [BCG: Y] or BCG naïve [BCG: N] at baseline), years since BCG vaccination (grouped as 1 to 9, 10 to 19, or 20 to 29 years, or “never”), gender, and monocyte-to-lymphocyte cell count ratio (ML ratio). The macaque population covariate was the colony of origin (Indian rhesus macaques; for cynomolgus macaques, Chinese, Mauritian, or Indonesian [see Table S2 and Fig. S2 in the supplemental material]). Rhesus macaques and cynomolgus macaques of the Indonesian and Mauritian genotypes were obtained from established UK breeding colonies. Chinese cynomolgus macaques were imported from a Home Office-approved breeding colony in China.

### Mathematical IS/ID model.

An ordinary differential-equation model was used to describe the IFN-γ response dynamics of two CD4^+^ T cell populations, transitional effector memory (36) and resting “central” memory cells, which are short- and long-lived, respectively (37–39) (Fig. 1). Briefly, cells were recruited into the transitional effector memory compartment at rate δ. A proportion (p) of transitional effector memory cells underwent apoptosis at rate μ_TEM_, and the remaining proportion (1 − p) transitioned to a central memory phenotype, where they stayed for the duration of the model run (170 days) (Fig. 1). Central memory cells are quiescent in the host until stimulated by an antigen (31); however, we considered them here to contribute to IFN-γ production, since the ELISPOT assay uses PPD to stimulate all potentially IFN-γ-secreting CD4^+^ T cells. To reflect this, the IFN-γ immune response predicted by the mathematical model was the sum of the number of transitional effector memory and central memory cell populations over time. We assumed that any nonzero responses at baseline were existing memory responses that had immediately reverted to the transitional effector memory phenotype in the presence of an antigen. Therefore, the initial transitional effector memory population (TEM_0_) was positive for those subjects. We assumed that the increases in the number of transitional effector memory and central memory cells did not occur immediately after vaccination but gradually increased over time due to immune processes such as vaccine antigen trafficking and presentation (31, 40). This increase then subsided, as T cell stimulation was assumed not to last indefinitely (31, 40–43). The recruitment of transitional effector memory cells over time was controlled in the model using the recruitment rate δ, which was a peaked curve specified using a gamma probability density function (PDF) distribution with parameters L, k, and h (Fig. 1).

### Analyses. (i) Analysis 1. Calibration of the model to IFN-γ data and exploration of model predictions for macaques and humans separately.

In analysis 1, the model was calibrated to the macaque and human data separately to quantify the dynamics of the IFN-γ response for each species. To do this, three parameters (the components of function δ: L, k, and h [Fig. 1]) and TEM_0_, the initial number of transitional effector memory cells, were estimated using the established method of nonlinear mixed effects modeling (NLMEM) (29) using the software *Monolix* v. 4.3.3 (44). Briefly, NLMEM uses maximum likelihood methods to estimate the model parameters that best describe the population mean response and the associated parameter variance which accounts for the within-population variation (for more details see reference 45). Evaluation of the model's ability to describe the data was conducted primarily by simulation-based, visual predictive check (VPC) plots (see the supplemental material for details); assessment of the precision of the estimated parameters using the relative standard error (RSE) and a goodness of fit measure (Bayesian Information Criteria [BIC]). A difference in BIC of >6 was considered a significant (*P* value < 0.05) effect (46) and a parameter RSE of <30% was considered a well-estimated parameter. The proportion of transitional effector memory cells that die (p) was assumed to be 0.925, as supported by literature (38) (Fig. 1; Table 1) and the parameter governing the mortality rate of transitional effector memory cells, μ_E_, was fixed after a scenario analysis was conducted (Table S3). Further tests required to establish the NLMEM framework are outlined in Tables S4 to S6.

### (ii) Analysis 2. Population covariate impact on within-population variation in model parameter estimates.

In analysis 2, we explored whether population covariates (i.e., subpopulations, such as different colonies) could reduce the within-population variation of the estimated parameters from that in analysis 1, and we thus established subpopulation models for macaques and humans separately. To do this, covariate-parameter relationships were tested and selected based on a forward-addition strategy and likelihood ratio test method (see the supplemental material for details). Once the appropriate covariate-parameter relationship was found, the subpopulation model was calibrated to the data and the subpopulation parameters estimated. We observed the change in the BIC values and within-population variation of model parameters from analysis 1 to analysis 2 as a result of accounting for the population covariates.

### (iii) Analysis 3. Which macaque subpopulations best predicted immune responses in different human subpopulations?

To evaluate which macaque subpopulations best predicted the immune responses in different human subpopulations, estimated parameters and parameter variances from the macaque subpopulation model (analysis 2) were fit to the human data (or human subpopulation data [analysis 2]). The subpopulation of macaques that best described the human data was defined as the model with the lowest BIC.

### (iv) Analysis 4. Predicted numbers of TEM and resting CM cells over time.

The calibrated mathematical model was then used to predict the number of transitional effector memory (36) and resting central memory cells over time. These dynamics were not measured empirically.

## Supplementary Material

Supplemental material
