# Supplementary material for: Using Data from Macaques To Predict Gamma Interferon Responses after Mycobacterium bovis BCG Vaccination in Humans: a Proof-of-Concept Study of Immunostimulation/Immunodynamic Modeling Methods
Source: Clin Vaccine Immunol. 2017 Mar 6;24(3):e00525-16. doi: 10.1128/CVI.00525-16 (PMC5339646; doi:10.1128/CVI.00525-16)
Supplement: Supplemental material [file supp_24_3_e00525-16__index.html]

Supplemental material 

# Using Data from Macaques To Predict Gamma Interferon Responses after Mycobacterium bovis BCG Vaccination in Humans: a Proof-of-Concept Study of Immunostimulation/Immunodynamic Modeling Methods

## Supplemental material

- Supplemental file 1 -

  Additional methods. Table S1. Human demographics. Table S2. Macaque demographics. Fig. S1. Longitudinal IFN-γ responses for analysis for 55 human participants. Fig. S2. Number of IFN-γ-secreting CD4<sup>+</sup> T cells per million PBMCs over time as measured by the ELISPOT assay in macaques. Analysis S1. Model calibration to IFN-γ data and exploration of model predictions for macaque and humans, separately. Table S3. Scenario analysis for parameter μ<sub>TEM</sub> in macaques and humans. Table S4. Results of comparing residual error models using the Monolix in-built tool. Table S5. Tests for random effects correlations for macaques and humans. Analysis S2. Population covariate impact on within-population variation in model parameter estimates. Analysis S3. Which macaque subpopulations best predicted immune responses in different human subpopulations? Additional results. Table S6. Residual error model estimated parameters for a combined residual error model for macaques and humans. Fig. S3. Residual (difference between data and total cells as predicted by the model) plots for macaque predicted total responses. Fig. S4. Residual (difference between data and total cells as predicted by the model) plots for human predicted total responses..Fig. S5. Empirical data versus predicted total IFN-γ responses for macaques (A) and humans (B). Fig. S6. Data, predicted total number of T cells secreting IFN-γ, predicted number of transitional effector memory (TEM) cells, and predicted number of resting central memory (CM) cells, over time. Fig. S7. Prediction distribution plot for macaques (A) and humans (B). Table S7. <i>P</i> value results of applying the nonparametric Kruskal-Wallis and post-hoc Dunn test (for more than two groups) on individual macaque estimated parameters from Analysis S1, with colony as the predictor. Fig. S8. Boxplot of individual macaque estimated parameters from Analysis S1 by colony. Table S8. Forward stepwise addition method for selecting a subpopulation model for colony in macaques. Table S9. Results of applying the Wilcoxon test on individual human estimated parameters from Analysis S1, with gender as the predictor. Fig. S9. Boxplot of individual human estimated parameters from Analysis S1 by gender. Table S10. Results of applying linear regression on individual human estimated parameters from Analysis S1, with ML ratio as the predictor. Fig. S10. Scatter plots of individual human estimated parameters from Analysis S1 against ML ratio. Table S11. <i>P</i> value results of applying the nonparametric Kruskal-Wallis and post-hoc Dunn test (for more than two groups) with a Bonferroni correction on individual human estimated parameters from Analysis S1, with BCG history as the predictor. Fig. S11. Boxplot of individual human estimated parameters from Analysis S1 by BCG history. Table S12. Results of applying the Wilcoxon test on individual human estimated parameters from Analysis S1, with BCG status as the predictor. Fig. S12. Boxplot of individual human estimated parameters from Analysis S1 by BCG status. Table S13. Forward stepwise addition method for selecting a covariate model for BCG status in humans. Fig. S13. Visual predictive check plots for all colonies of macaque. Fig. S14. Visual predictive check plots for BCG: N and BCG: Y humans. Fig. S15. Residual (difference between data and total cells as predicted by the model) plots for macaque predicted total responses stratified by colony. Fig. S16. Macaque observed versus predicted IFN-γ total responses stratified by colony. Fig. S17. Residual (difference between data and total cells as predicted by the model) plots for human predicted total responses stratified by BCG status. Fig. S18. Human observed versus predicted IFN-γ responses stratified by BCG status. Fig. S19. Prediction distribution plot for all colonies of macaque. Fig. S20. Prediction distribution

  PDF, 3.6M
