# Supplementary material for: Using Data from Macaques To Predict Gamma Interferon Responses after Mycobacterium bovis BCG Vaccination in Humans: a Proof-of-Concept Study of Immunostimulation/Immunodynamic Modeling Methods
Source: Clin Vaccine Immunol. 2017 Mar 6;24(3):e00525-16. doi: 10.1128/CVI.00525-16 (PMC5339646; doi:10.1128/CVI.00525-16)
Supplement: Supplemental material [file CVI.00525-16_zcd999095453s1.pdf]

Additional Material

Contents

1 Additional Methods 2

Data 2

Mathematical Immunostimulation/Immunodynamic (IS/ID) Model 6

Analyses 6

Analysis 1: Model calibration to IFN-γ data and exploration of model predictions for macaque and humans, separately 6

Analysis 2: Population covariate impact on within-population variation in model parameter estimates 8

Analysis 3: Which macaque subpopulations best predicted immune responses in different human subpopulations? 9

2 Additional Results 10

Analysis 1: Model calibration to IFN-γ data and exploration of model predictions for macaque and humans, separately 10

Analysis 2: Population covariate impact on within-population variation in model parameter estimates 17

Analysis 3: Which macaque subpopulations best predicted immune responses in different human subpopulations? 32

3 Additional Discussion 40

1 Additional Methods

Data

Human demographic data and spaghetti plot can be found in Table S1 and Figure S1, respectively.

Total population	55
Age; median (range)	25 (18, 55)
Baseline-BCG status	BCG: N= 30, BCG: Y=25
Gender	M=19, F=36
Time since BCG vaccination	
1 to 9	7 (M = 2)
10 to 19	10 (M = 6)
20 to 29	8 (M = 3)
30+	-
Never	30 (M = 8)
ML ratio; median (range)	0.26 (0.07, 0.56)

Table S1. Human demographics

The available data were on HIV negative and Mtb naïve participants (see [1-3] for HIV and Mtb latency testing procedures). Data on haematological parameters were based on routine laboratory haematology testing at baseline and only those participants with values within normal limits were included in clinical trials. IFN-γ response was measured using a standardized ex vivo IFN-γ Enzyme-Linked ImmunoSpot (ELISPOT) assay which quantifies IFN-γ secreting CD4+ T cells as spot forming units (SFU) per million peripheral blood mononuclear cells (PBMCs) using PPD as a stimulant. The same ELISPOT method including plates, antibody kits, antigens, developing reagents, washing method, ELISPOT reader and ELISPOT counting method were used in all the data collection. As these BCG studies were conducted as part of a series of Phase I clinical trials with MVA85A all lab protocols and lab reagents were harmonized as far as possible. For the IFN-γ ELISPOT assay 300,000 PMBC per well were performed in duplicate and the results were averaged. Incubation time was 18 hours. For the exact laboratory methodology see [1-3] .

The four covariates included in this analysis, gender, BCG vaccination history at baseline and baseline ML ratio. For details on how BCG-vaccination history was determined see original trial methods [1-3]. BCG vaccination history was categorised into “never” and 10 year time-periods since vaccination with

the reference group as 1 to 9 years since BCG vaccination. Age was not included as a covariate as it was colinear with BCG vaccination history.

For macaques, these can be found in Table S2 and Figure S2.

**Species (% of total animals)**	**Colony (% of total animals)**
Rhesus, n= 58 (72%)	India, n= 58 (72%)
Cynomolgus, n= 23 (28%)	Mauritian, n=12 (15%)
	Chinese, n=6 (8%)
	Indonesian, n= 5 (6%)

Table S2: Macaque demographics

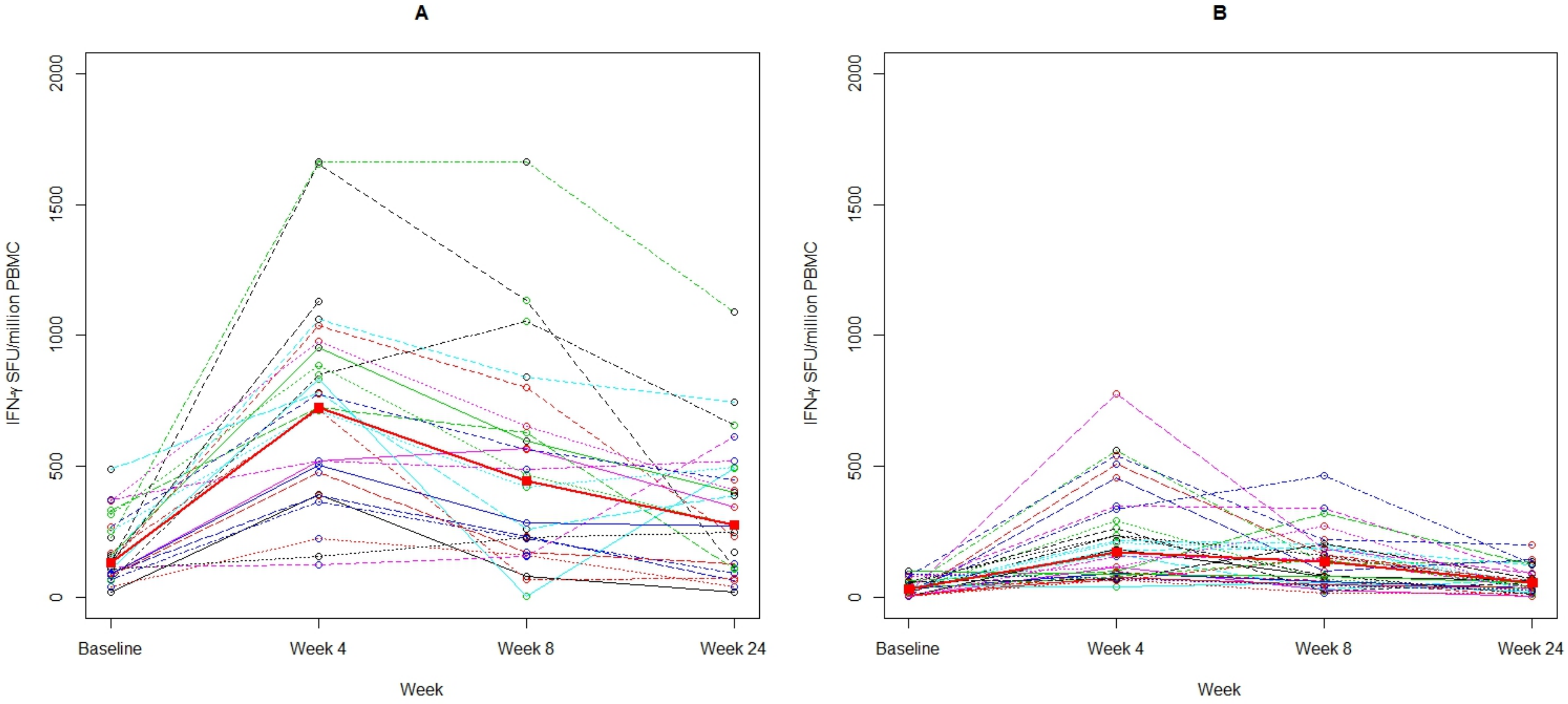

Figure S1. . Longitudinal IFN-γ responses for analysis for 55 human participants. Baseline-BCG vaccinated (A) and baseline-BCG naive (B). The bold line represents the median values of each group at each time point. X-axis is not to scale. Abbreviations: IFN-γ = Interferon gamma ; SFU = spot forming unit ; PBMC = peripheral blood mononuclear cells

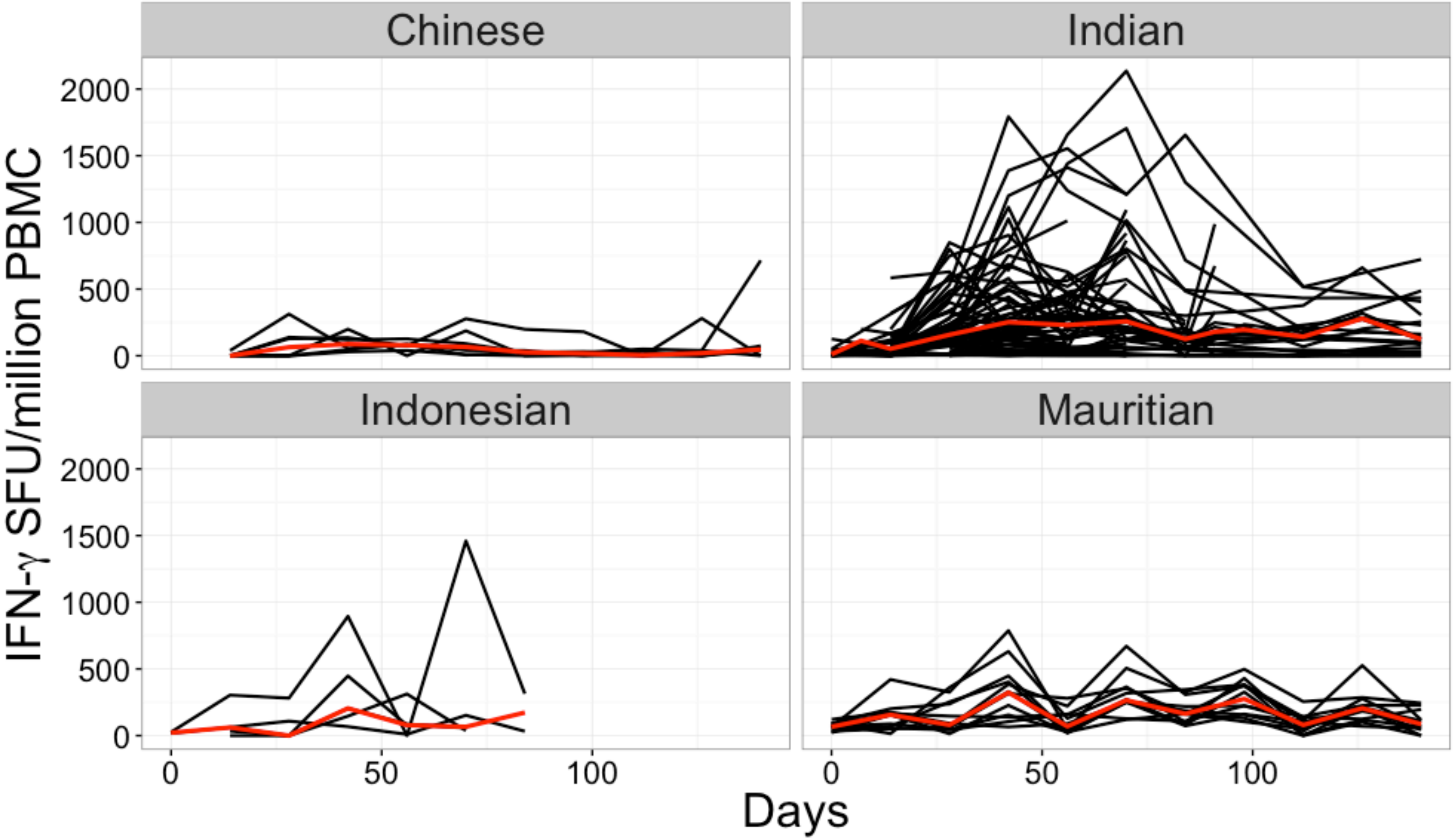

Figure S2: Number of IFN-γ secreting CD4+ T cells per million PBMCs over time as measured by the ELISPOT assay in macaques. Data is shown for each colony separately, Chinese, Indonesian and Mauritian cynomolgus macaques and Indian rhesus macaques. The red line indicates median responses.

Mathematical Immunostimulation/Immunodynamic (IS/ID) Model

The equations for the IS/ID two-compartmental in Figure 1 are as follows:

$$\frac{dTEM}{dt} = \delta - p\mu_{TEM}TEM - (1-p)\mu_{TEM}TEM \quad (1)$$

$$\frac{dCM}{dt} = (1-p)\mu_{TEM}TEM \quad (2)$$

Where TEM represents the transitional effector memory (TEM) cell population, CM, the resting central memory (CM) cell population, t, the time in days and parameters outlined in Figure 1. The equation for the recruitment of the TEM cell population, δ, is:

$$\delta = L * \frac{\left(1/h\right)^{k}}{\Gamma(k)} * time^{(k-1)} * e^{-\left(\frac{1}{h} * time\right)} \quad (3)$$

Where L, h and k are the gamma pdf parameters outlined in Figure 1.

Analyses

Analysis 1: Model calibration to IFN-γ data and exploration of model predictions for macaque and humans, separately

Visual Predictive Check (VPC) plot

The visual predictive check plot (VPC) is a simulation based diagnostic tool for assessing the appropriateness of the proposed mathematical model to describe the empirical data. This is done by comparing data simulated using the model and estimated population mean parameters and associated variances, to the empirical data distribution [4]. To construct the VPC, the mathematical model (Figure 1) is calibrated to the dataset in question (e.g. the entire human population data) and the resulting estimated parameters and associated variances (Table 1) are used to simulate a theoretical population dataset, equivalent to the size of the population in question (e.g. N=55 for the entire human population). This procedure is repeated 500 times and the 10th, 50th and 90th percentiles of each simulated population dataset are recorded and the ranges of these percentiles are plotted. If the model is appropriate to represent the data, when the observed percentiles are plotted alongside the VPC, they should fall in the bounds of the simulated percentile ranges. Figure 2 outlines displays the VPC plots for the human and macaque model predictions.

Scenario analysis for parameter μ_{TEM} (per day)

	Macaque	Human
Param μ_{TEM} (per day)	BIC	BIC
1/2	7269.01	2825.22
1/4	7259.65	2803.31
1/6	7254.20	2791.84
1/8	7251.55	2785.49
1/10	**7248.89**	2780.97
1/12	7254.49	**2778.53**
1/14	7259.81	2780.80
1/16	7263.44	2782.09

Table S3: Scenario analysis for parameter μ_{TEM} in macaques and humans

Table S3 summarises the scenario analysis of parameter μ_{TEM} in macaques and humans. In macaques the value of 1/10 for μ_{TEM} resulted in the lowest BIC value, however there was no significant difference in the BIC for the values μ_{TEM} from 1/6 to 1/12 (shaded) (see [5] for significance associated with difference of BIC values). Similarly, in humans the value of 1/12 for μ_{TEM} resulted in the lowest BIC value, with no significant difference between values of 1/10 to 1/16 (shaded).

Residual Error (RE) Model

Table S4 outlines the results of the RE model comparison for macaques and humans separately using BIC as an assessment of fit.

Error model	Model Description	Macaque	Human
		BIC	BIC
Constant	Y = f+a*e	7753.10	2895.72
Proportional	Y = f+b*f*e	-	2780.65
Combined	Y = f+(a+b*f)*e	7248.89	2776.66

Table S4: Results of comparing residual error models using Monolix in-built tool. Definitions: Y = observation, f = model prediction, a,b= scalars to be determined during parameter estimation process, e = Normally distributed random variable N(0,1).

The BIC for the human residual error model indicate that a combined model best represented the residual error in the data, however the proportional or combined model were not significantly different with respect to calibration to the data.

The same comparisons were made for the macaque dataset, however when a RE model without an additive term was applied (e.g. a proportional model), the parameter TEM_0 was poorly estimated,

potentially due to a lack of data at time=0. Therefore, the BIC was compared between constant and combined RE models and combined was chosen.

The estimated values for the residual error model for macaque and human can be found in Table S6.

Test for random effects correlations

Results for the pairwise test for random effects correlations for human and macaques are shown in Table S5.

Combination tested	Macaque BIC	Macaque Diff to "none" (BIC)	Macaque Decision to include	Human BIC	Human Diff to "none" (BIC)	Human Decision to include
None	7253.68	-	-	2779.40	-	-
TEM_0 & L	7252.74	0.94	No	2788.21	8.81 (higher)	No
TEM_0 & k	7256.89	3.21 (higher)	No	2782.02	2.62 (higher)	No
TEM_0 & h	7258.99	5.1 (higher)	No	2778.45	0.95	No
L & k	7256.11	2.2	No	2784.77	5.37 (higher)	No
L & h	7257.62	3.73 (higher)	No	2778.35	1.05	No
k & h	7218.08	35.6	No*	2787.57	8.17 (higher)	No

Table S5: Tests for random effects correlations for macaques and humans

All BIC values in Table S5 were non-significantly different from no random effects correlations in the macaque population except for when parameters k and h were correlated. *However, applying this correlation meant that some parameters could not be accurately estimated (RSE% was NA) so it was not included. In the human population, all BIC values were either non-significantly lower, or higher than the model with no random effects correlations, so no correlations were considered necessary to apply in further analyses.

Analysis 2: Population covariate impact on within-population variation in model parameter estimates

Associations between population covariates and individual estimated parameters (from analysis 1) were conducted in R [6] using graphical plots and non-parametric rank tests for each species separately. The non-parametric rank tests conducted to establish parameter-covariate relationships are as follows. For categorical covariates with 2 levels (BCG status and gender in humans) the Wilcoxon test was applied. For categorical covariates with 2+ categories (BCG vaccination history in human and colony in macaques) a kruskal-Walllis followed by a Dunn post-hoc test with a Bonferroni correction was applied. For continuous covariate, ML ratio, linear regression was applied.

If a significant association (p-value<0.05) was found between model parameters and a covariate, a forward stepwise addition strategy was used in Monolix to establish a subpopulation-model. Here, parameter-covariate relationships were added to the subpopulation -model one at a time and the likelihood ratio test (LRT) was used to assess if the addition improved the fit of the covariate-model.

The parameter-covariate relationship was multiplicative, for example, the population estimation of the initial transitional effector memory cells (TEM_0) in accounting for BCG status was modelled by $TEM_{0BCG:N}=TEM_{0BCG:Y}*e^{\alpha}$, where $TEM_{0BCG:Y}$ is the value for TEM_0 for those in the BCG:Y subpopulation (the reference subpopulation) and α is the exponentiated scalar of this value to represent changes in TEM_0 for those in the BCG:Y subpopulation. The covariate effects (α's) are estimated in the NLMEM analysis alongside the associated p-values, but the value for the subpopulation parameter (left hand side of above equation) is reported in the results.

Analysis 3: Which macaque subpopulations best predicted immune responses in different human subpopulations?

In order to assess the calibrated macaque subpopulation parameters ability to describe the human data in Monolix, it was necessary to provide one parameter to estimate. To achieve this, all subpopulation parameters were fixed at their calibrated value except for parameter L, which was allowed to vary within a minimal range of [calibrated value-1, calibrated value+1]. This provided a parameter to estimate to provide a BIC value but did not substantially change the calibrated macaque subpopulation parameters from analysis 2. The BIC values for this analysis are reported in Figure 4 in the main paper.

2 Additional Results

Analysis 1: Model calibration to IFN-γ data and exploration of model predictions for macaque and humans, separately

Estimates for the residual error model parameters

	Macaque				Human			
	**All (analysis 1)**		**Covariate (analysis 2)**		**All (analysis 1)**		**Covariate (analysis 2)**	
	**Estimated Value**	**RSE (%)**	**Estimated Value**	**RSE (%)**	**Estimated Value**	**RSE (%)**	**Estimated Value**	**RSE (%)**
Additive contribution (cells)	5.37	90	5.51	87	3.79	65	6.04	43
Proportional contribution (% of predicted response)	61	10	61	9	42	10	39	10

Table S6: Residual error model estimated parameters for a combined residual error model for macaques and humans.

The estimates for the combined residual error model parameters for both macaques and humans for analysis 1 and 2 are in Table S6. The RSE for the additive component of the residual error model is high in both cases.

Diagnostic plots

Additional diagnostic plots for analysis 1 can be found in S3-S7.

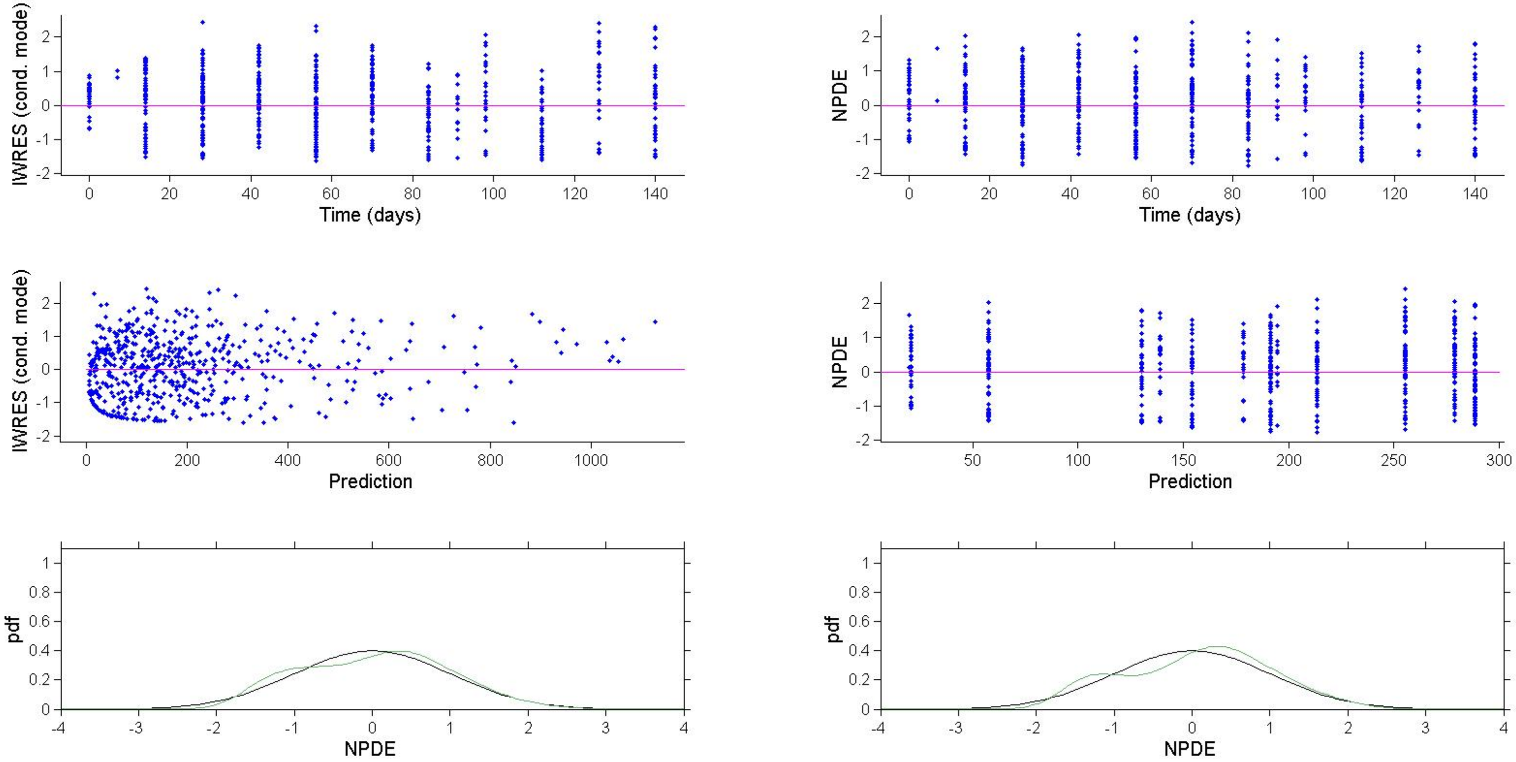

Figure S3: Residual (difference between data and total cells as predicted by the model) plots for macaque predicted total responses. The first row shows the individual weighted residuals (IWRES) and normalised prediction distribution errors (NPDE) using simulated individual parameters against time. The second row shows the residual error against the prediction. The bottom rows show the distribution of the residuals compared to a Gaussian pdf curve so assess the normality of the residuals.

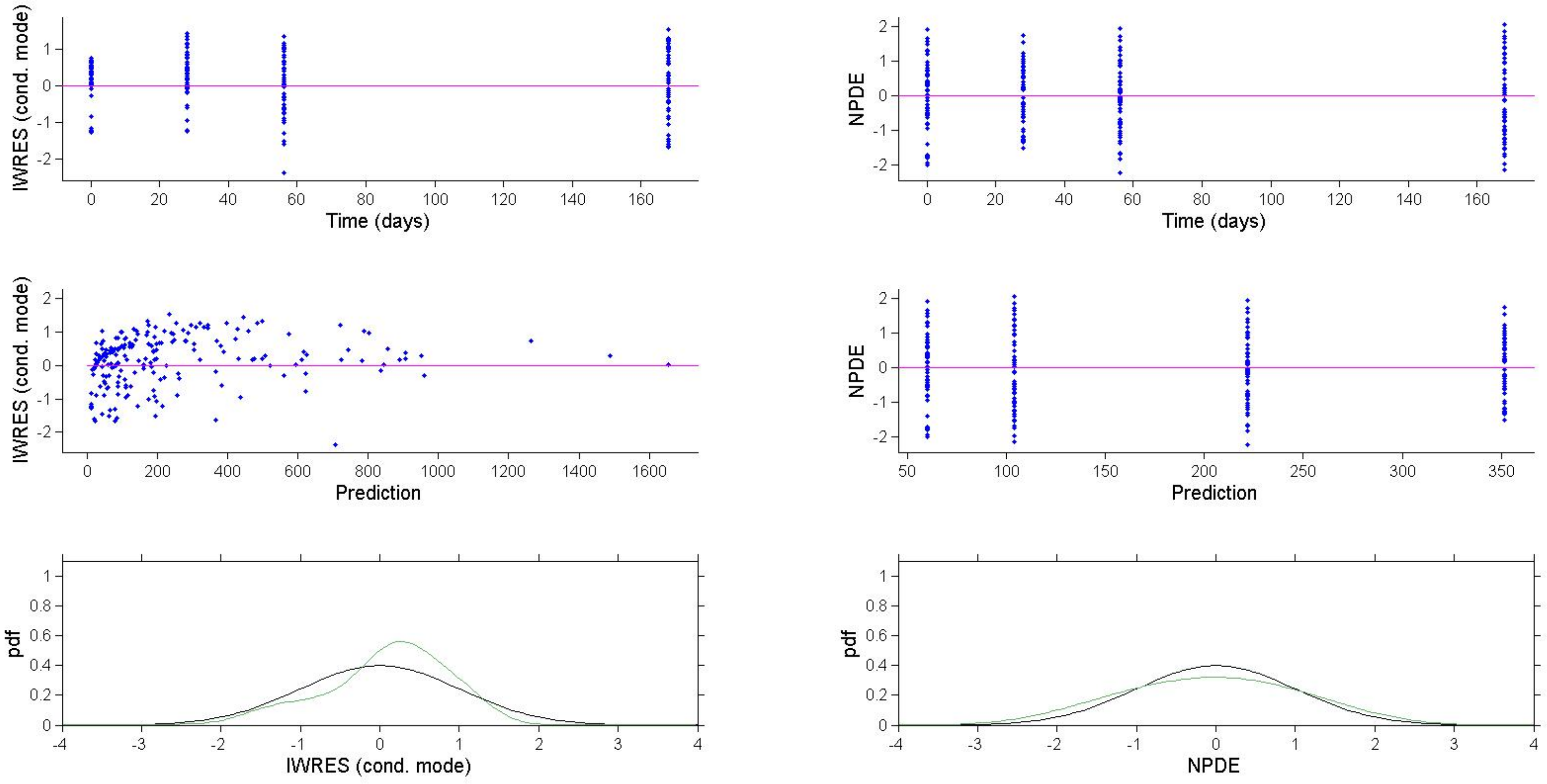

Figure S4: Residual (difference between data and total cells as predicted by the model) plots for human predicted total responses. The first row shows the individual weighted residuals (IWRES) and normalised prediction distribution errors (NPDE) using simulated individual parameters against time. The second row shows the residual error against the prediction. The bottom rows show the distribution of the residuals compared to a Gaussian pdf curve so assess the normality of the residuals.

Figure S3 shows the residual plots for the macaque total cell predictions in analysis 1. The residuals seem to be normally distributed, although the IWRES by time and the prediction pdf (bottom row) show slight model under prediction. This is most apparent at time points 84 and 112. However, this may be a result of the large variation in the data between time points (particularly for day 84 and 112), which the model is unable to accommodate. Figure S4 shows the residual plots for the human total cell predictions in analysis 1. Residual error in this case seem to approximate a normal distribution, however there appears to be slight under prediction by the model, particularly at day 0 and 28 (IWRES plot, top row). Despite this, the VPC in Figure 2 indicates that the model is still

an adequate prediction of the data, for both species. The observed versus predicted response plots in Figure S5 also reflect the discrepancy between data and model total cell predictions for population (left column) and individual (right column) data for A. macaques and B. humans.

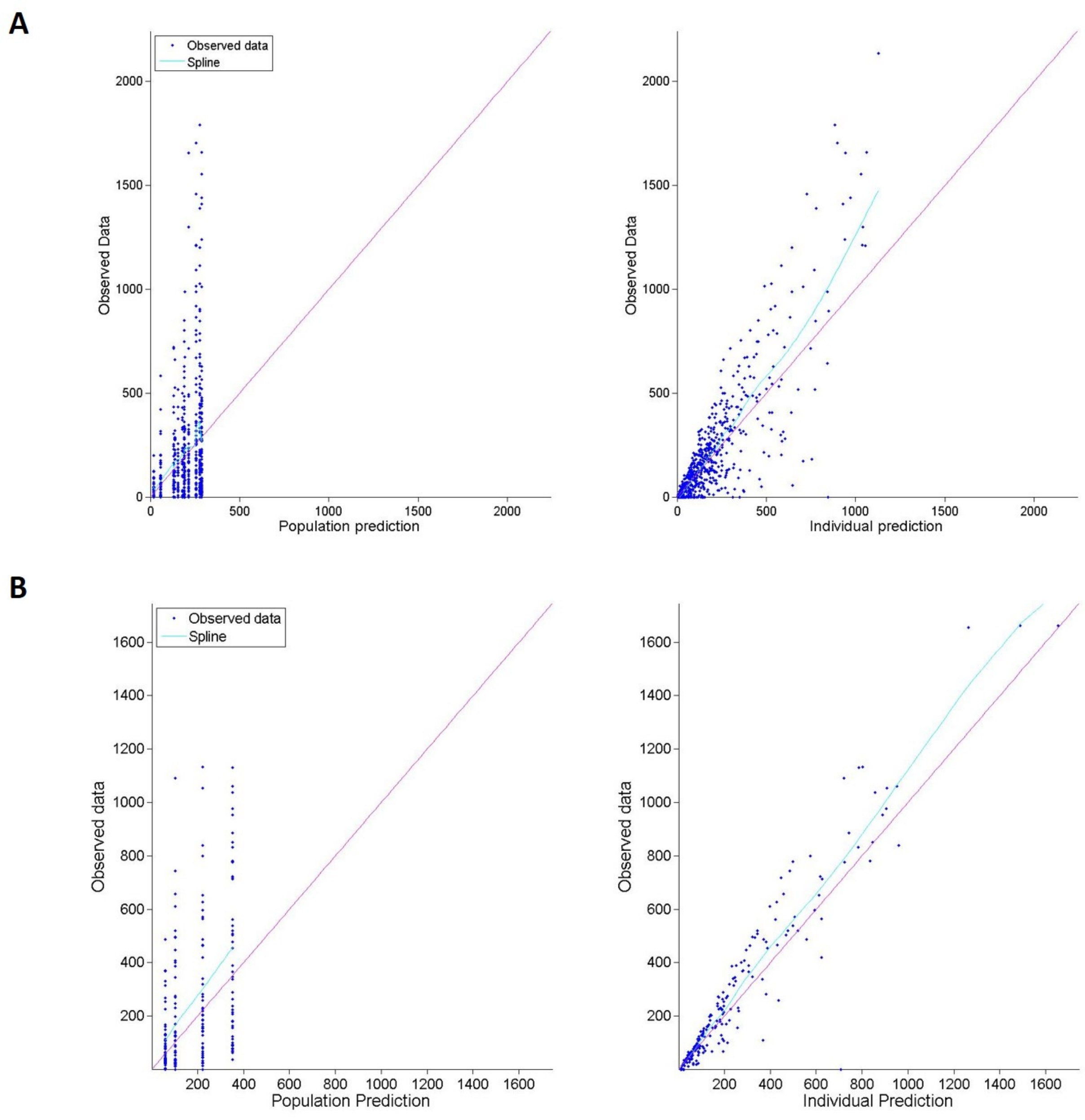

Figure S5: Empirical data versus predicted total IFN-γ responses for A. macaques and B. humans

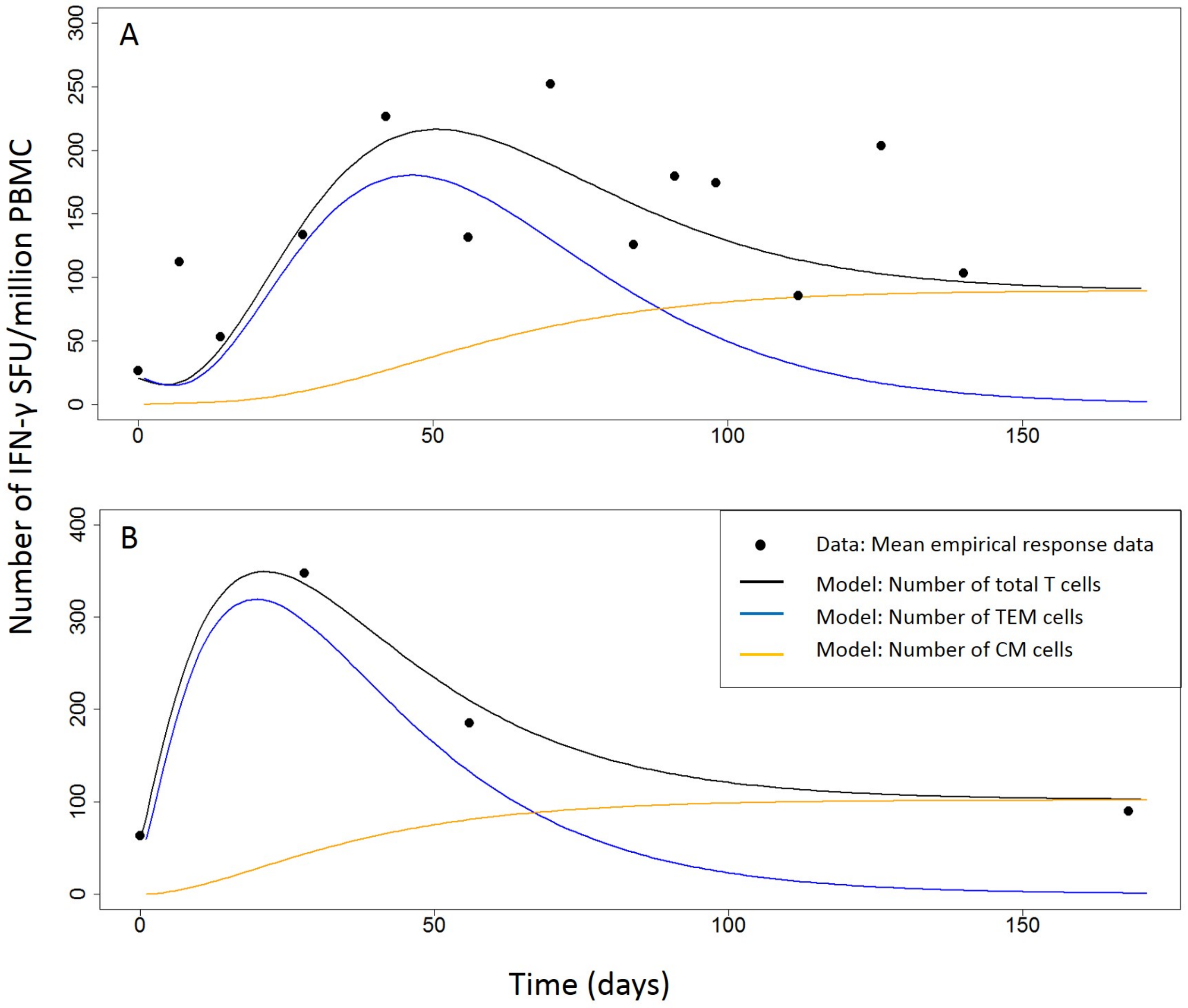

Figure S6: Data (black points), predicted total number of T cells secreting IFN-γ (black line), predicted number of transitional effector memory (TEM) cells (blue line), and predicted number of resting central memory (CM) cells (orange line), over time. Model predictions use the estimated parameters from Table 1 for the A) macaque and B) human populations.

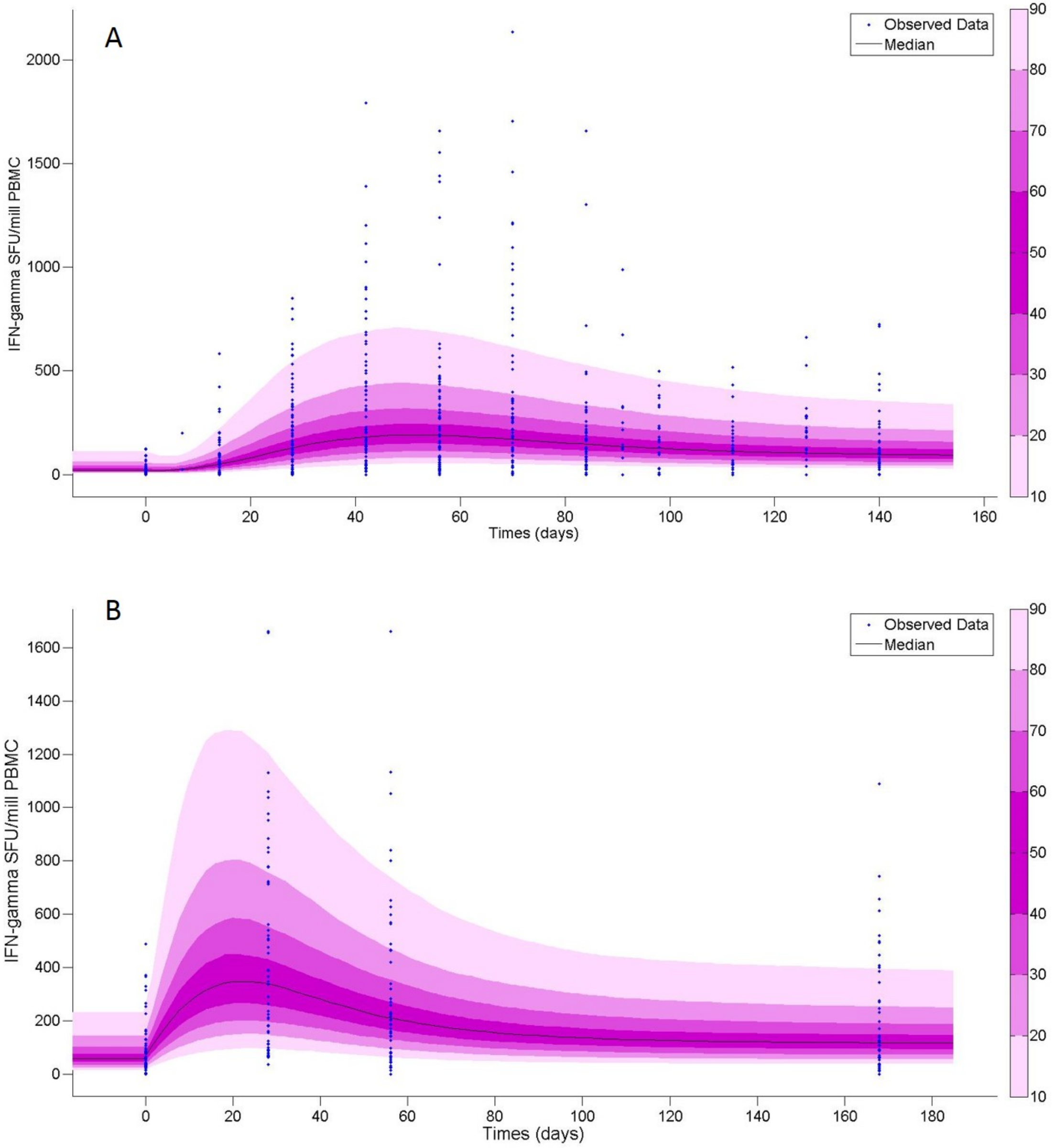

Figure S7. Prediction distribution plot for A. macaques and B. humans. The black points represent the empirical data. The bands represent the 10th to 90th percentiles of the theoretical predictions using the estimated population parameters and associated variation for analysis 1 (Table 1). The black line shows the median total cell response prediction

Analysis 2: Population covariate impact on within-population variation in model parameter estimates

Non-parametric rank test in R on individual macaque estimated parameter values from Analysis 1

Colony

TEM_0				L			
	Cyn: Chi	Rhe: Ind	Cyn: Indo		Cyn: Chi	Rhe: Ind	Cyn: Indo
Rhe: Ind	NS			Rhe: Ind	NS		
Cyn: Indo	**S**	NS		Cyn: Indo	NS	NS	
Cyn: Maur	**S**	**S**	**S**	Cyn: Maur	**S**	NS	**S**
k				h			
	Cyn: Chi	Rhe: Ind	Cyn: Indo		Cyn: Chi	Rhe: Ind	Cyn: Indo
Rhe: Ind	NS			Rhe: Ind	NS		
Cyn: Indo	NS	NS		Cyn: Indo	NS	NS	
Cyn: Maur	**S**	NS	NS	Cyn: Maur	NS	NS	NS

Table S7: p-value results of applying the non-parametric Kruskal-Wallis and post-hoc Dunn test (for more than two groups) on individual macaque estimated parameters from analysis 1 with colony as the predictor. Abbreviations: Cyn: chi = cynomolgus macaques of Chinese origin, Cyn: Maur = cynomolgus macaques of Mauritian origin, Cyn: Indo= cynomolgus macaques of Indonesian origian, Rhe: Ind = Rhesus macaques of Indian origin. NS equates to non-significant (p-value>0.05), S equates to significant (p-value<0.05).

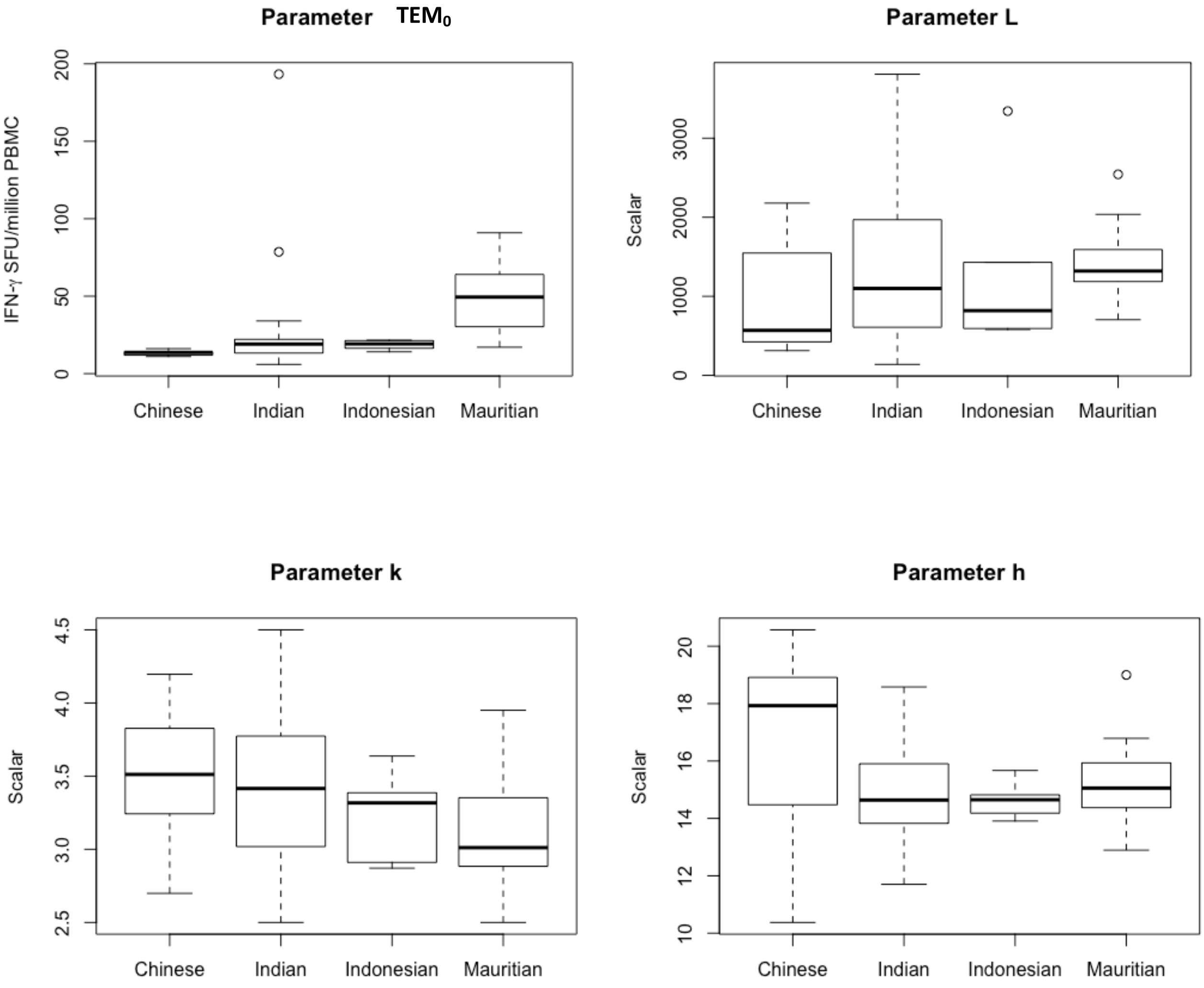

Figure S8: Boxplot of individual macaque estimated parameters from analysis 1 by colony

Table S7 and Figure S8 show that there is significant difference on the individual macaque estimated parameters TEM_0, L and k between the Chinese, Mauritian, Indonesian and Indian macaques. The colony covariate will be added to the covariate model for macaques in analysis 2.

Forward stepwise addition method for selecting macaque covariate model

Model #	Parameter(s)	-2LL	Diff in -2LL (*from Model #)	0.05 level significant? (Chi^2 test 1 d.f.: crit val = 3.84)
1	TEM_0	7189.96	-	-
2	L	7206.53	-	-
3	k	7209.26	-	-
4	h	7222.45		
3	TEM_0+L	7183.75	6.21 (*1)	Yes
4	TEM_0+k	7185.89	4.07 (*1)	Yes
5	TEM_0+h	7199.01	+9.05 (*1)	No

5	TEM_0+L+k	7177.55	6.2 (*3)	Yes

Table S8: Forward stepwise addition method for selecting a subpopulation-model for colony in macaques. -2LL values are taken from running in Monolix with colony applied to the parameter. Difference in -2LL from the nested model (indicated with a *) is calculated and significance is assessed by a chi squared distribution for one degree of freedom (for a p-value of 0.05, this is a critical value of 3.84).

Non-parametric rank test in R on individual human estimated parameter values from Analysis 1

Gender

Parameter	Wilcoxon test p-value
TEM_0	0.45
L	0.26
k	0.31
h	0.14

Table S9: Results of applying the Wilcoxon test on individual human estimated parameters from analysis 1 with gender as the predictor

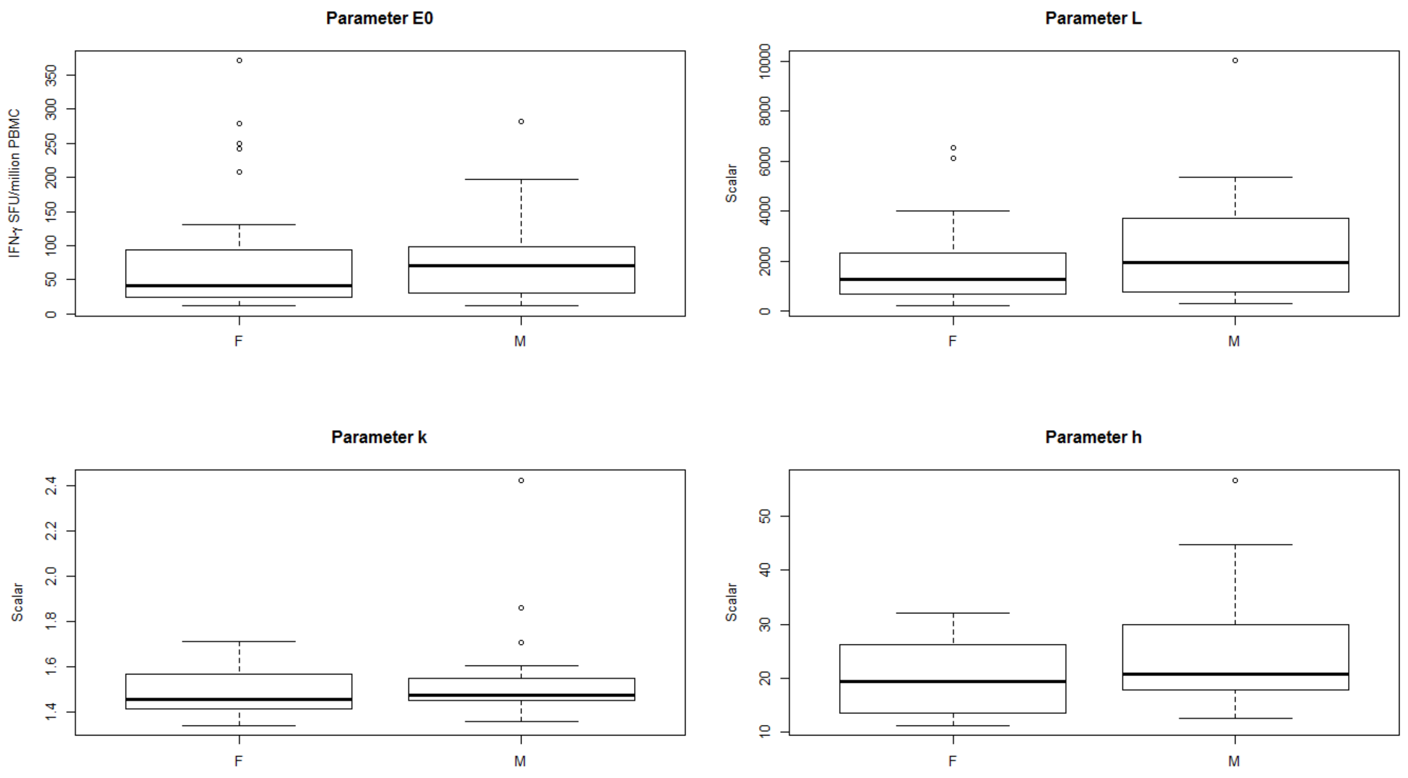

Figure S9: Boxplot of individual human estimated parameters from analysis 1 by gender, F=Female, M=Male

As gender did not significantly impact the individual human estimated parameters (Table S9 and Figure S9), it was not considered further in this work.

ML Ratio

Parameter	Linear regression slope parameter p-value

TEM_0	0.70
L	0.69
k	0.33
h	0.24

Table S10: Results of applying linear regression on individual human estimated parameters from analysis 1 with ML ratio as the predictor

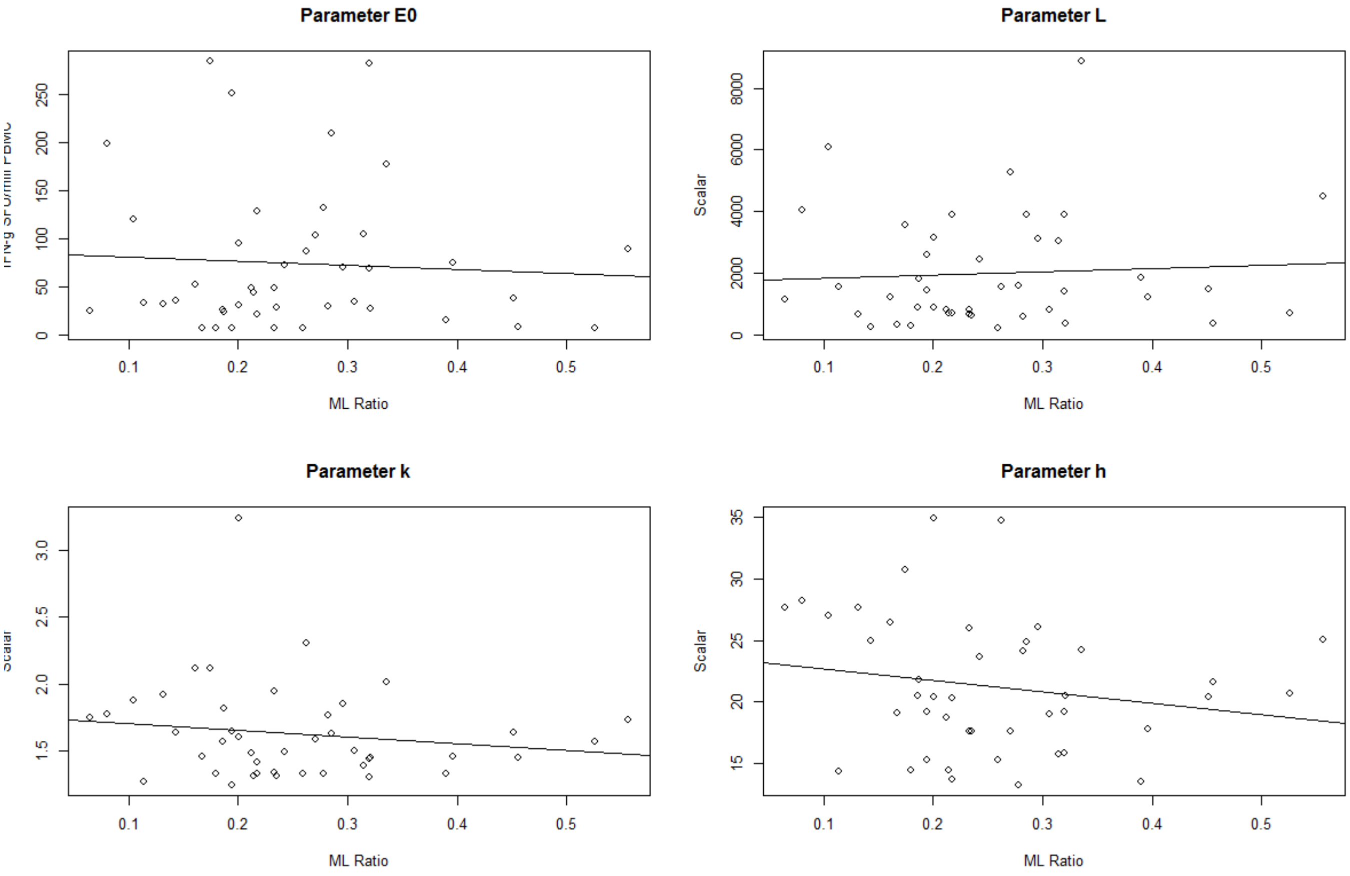

Figure S10: Scatterplots of individual human estimated parameters from analysis 1 against ML ratio

As ML ratio did not significantly impact the individual human estimated parameters (Table S10 and Figure S10), it was not considered further in this work.

BCG History

TEM_0	Never	10-19 yrs	1-9yrs
10-19 yrs	S		
1-9yrs	S	NS	
20-29 yrs	S	NS	NS

L	Never	10-19 yrs	1-9yrs
10-19 yrs	S		
1-9yrs	S	NS	
20-29 yrs	S	NS	NS

k	Never	10-19 yrs	1-9yrs
10-19 yrs	NS		
1-9yrs	NS	NS	
20-29 yrs	NS	NS	NS

h	Never	10-19 yrs	1-9yrs
10-19 yrs	NS		
1-9yrs	NS	NS	
20-29 yrs	NS	NS	NS

Table S11: p-value results of applying the non-parametric Kruskal-Wallis and post-hoc Dunn test (for more than two groups) with a Bonferroni correction on individual human estimated parameters from analysis 1 with BCG history as the predictor. NS equates to non-significant (p-value>0.05), S equates to significant (p-value<0.05).

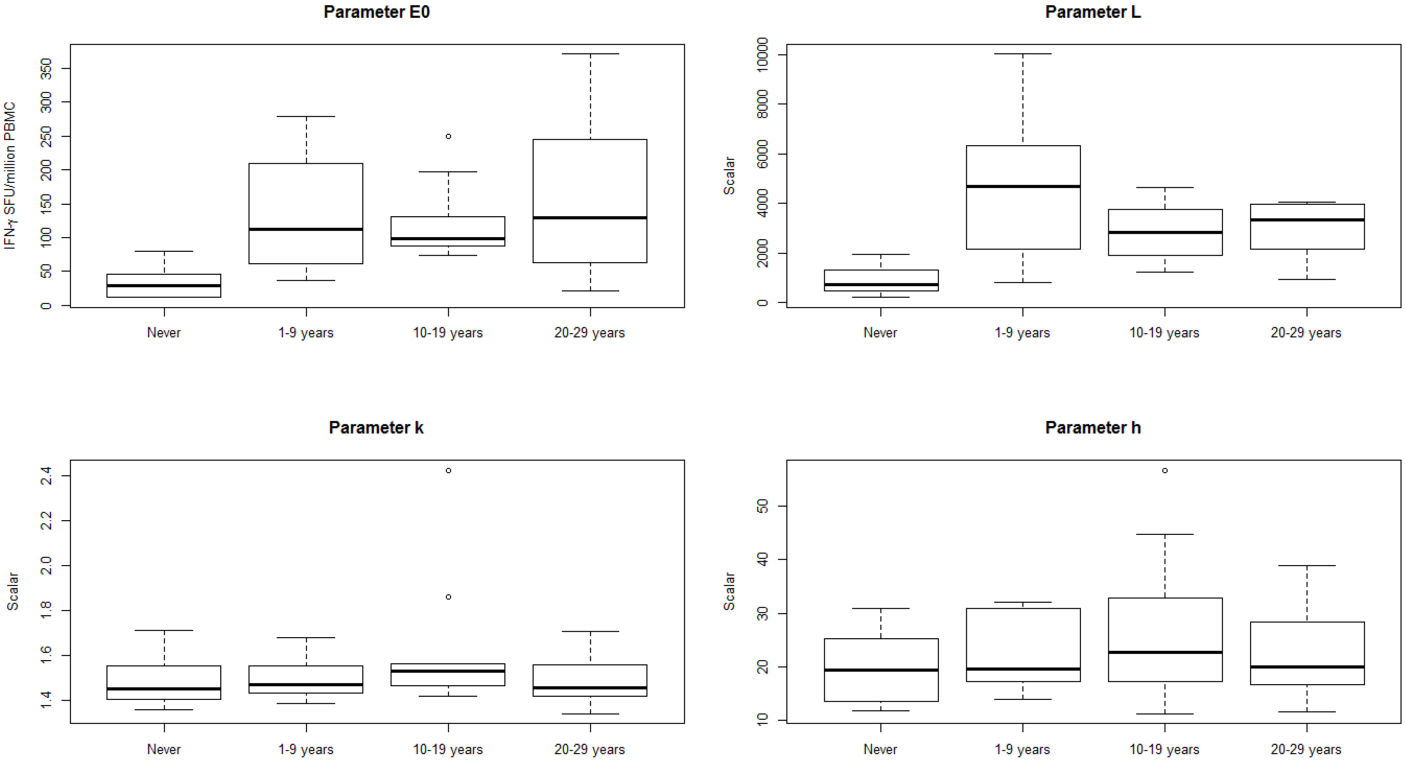

Figure S11: Boxplot of individual human estimated parameters from analysis 1 by BCG history

Table S11 and Figure S11 show that there is a significant difference on the individual estimated parameters between the "never" group, and the 1-9, 10-19 and 20-29 years since BCG vaccination groups, but not between the 1-9, 10-19 and 20-29 years since BCG vaccination groups. As such, these groups are considered as "BCG status", where 1+ years since BCG vaccination groups are aggregated into a BCG:Y group and the "never", BCG:N.

BCG Status

Parameter	Wilcoxon test p-value
TEM_0	2×10^{-10}
L	9.6×10^{-9}
k	0.31
h	0.13

Table S12: Results of applying the Wilcoxon test on individual human estimated parameters from analysis 1 with BCG status as the predictor

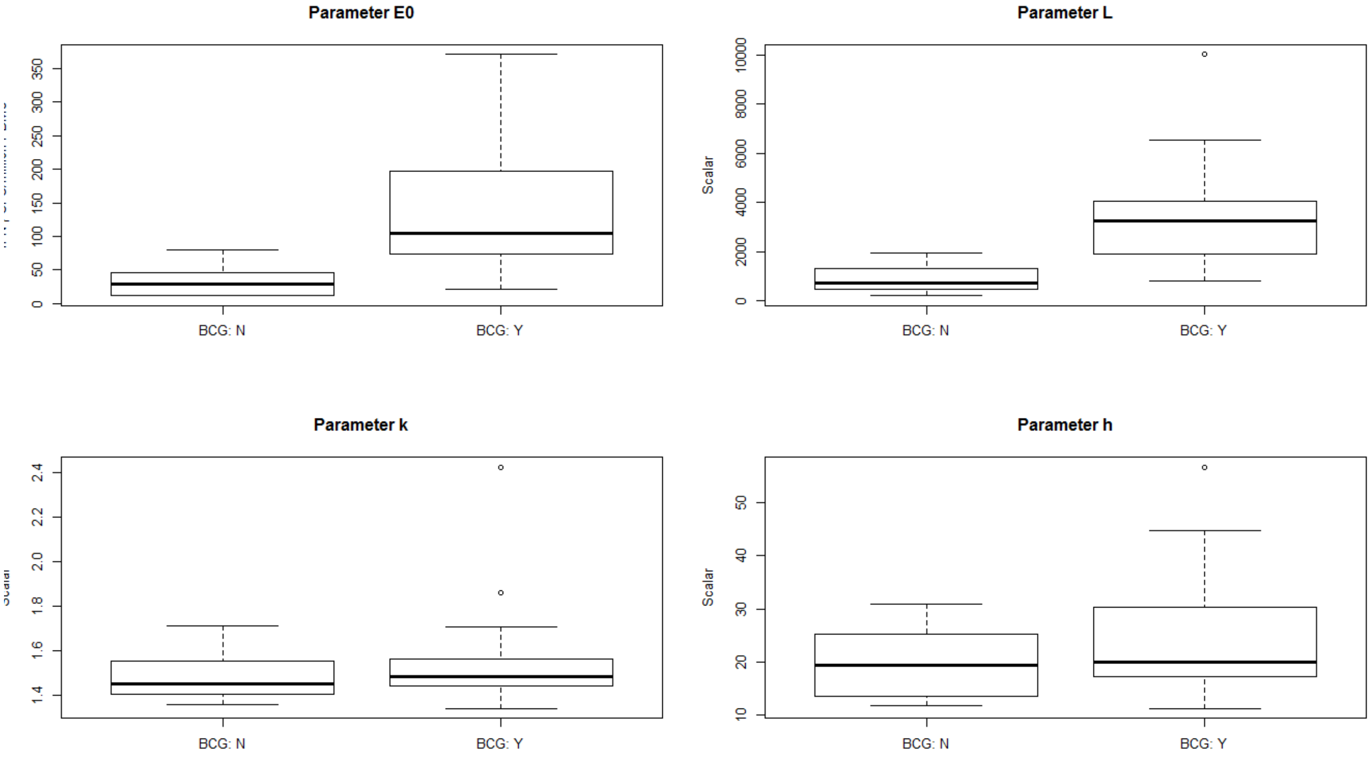

Figure S12: Boxplot of individual human estimated parameters from analysis 1 by BCG status

As BCG status significantly impacted the individual human estimated parameters (Table S12 and Figure S12), it was applied to parameters in Monolix.

Forward stepwise addition method for selecting human covariate model

Model #	Parameter(s)	-2LL	Diff in -2LL (*from Model #)	0.05 level significant? (Chi^2 test 1 d.f.: crit val = 3.84)
1	TEM_0	2698.00	-	-
**2**	**L**	**2697.36**	-	-
3	k	2739.45		
4	h	2737.22		
**5**	**L + TEM_0**	**2665.75**	**31.61 (*2)**	**Yes**
6	L + h	2694.01	3.35 (*2)	No
7	L + k	2696.85	0.51 (*2)	No
8	L+TEM_0+k	2657.75	8 (*5)	Yes
**9**	**L+TEM_0+h**	**2653.96**	**11.79 (*5)**	**Yes**
10	L+TEM_0+h+k	2651.54	2.42	No

Table S13: Forward stepwise addition method for selecting a covariate model for BCG status in humans. -2LL values are taken from running in Monolix with BCG status applied to the parameter. Difference in -2LL from the nested model (indicated with a *) is calculated and significance is assessed by a chi squared distribution for one degree of freedom (for a p-value of 0.05, this is a critical value of 3.84).

We chose the covariate-parameter relationship of L, TEM_0 and h for the BCG status covariate as, although it did not result in the lowest -2LL (see model 10, Table S13), parameters were better estimated.

Diagnostic plots
Additional diagnostic plots for the macaque and human subpopulation-models can be found in Figures S13-S20.

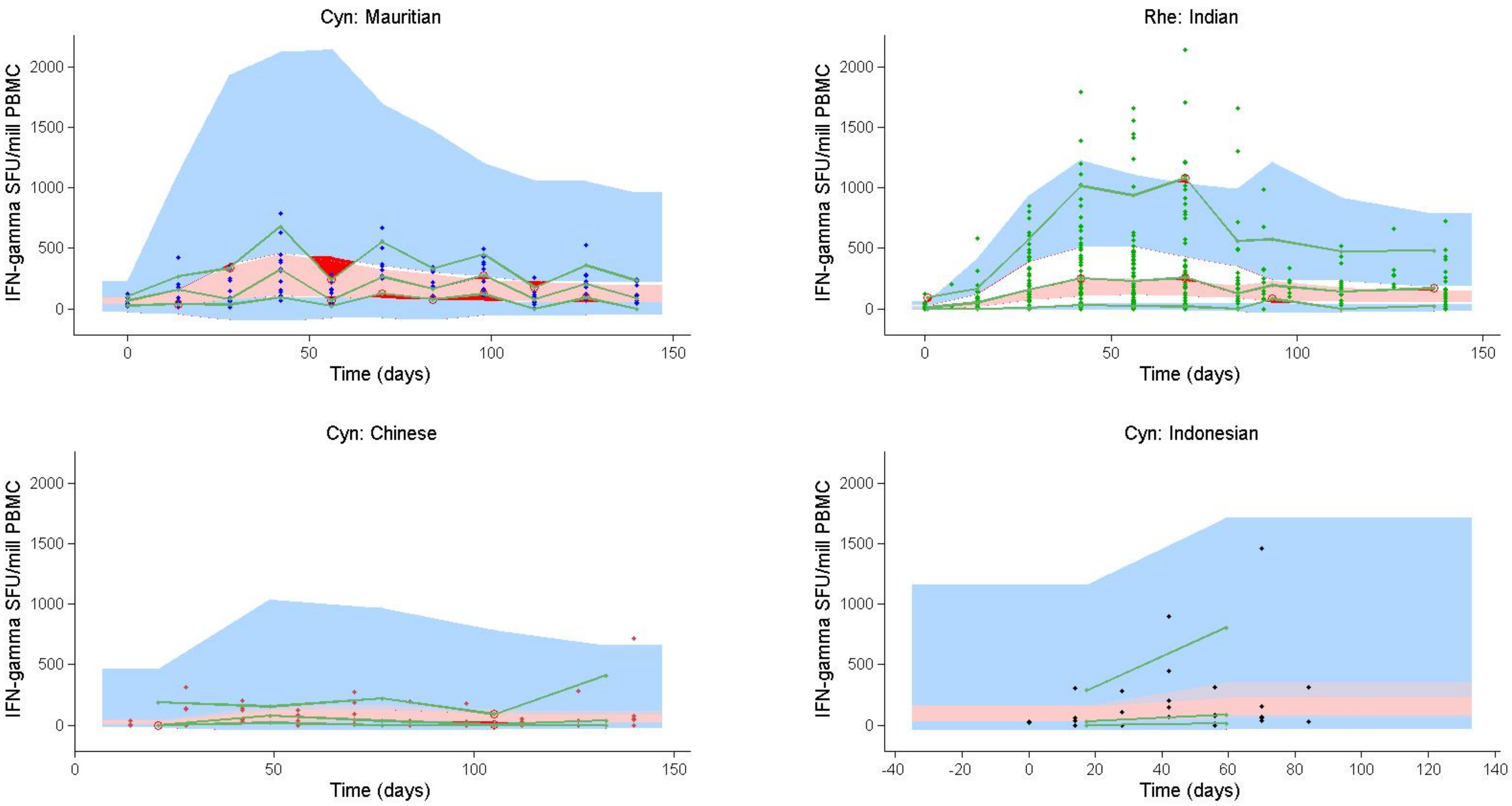

Figure S13: Visual predictive check plots for all colonies of macaque. Points represent the empirical data. Blue regions represent the ranges of the 90th and 10th percentiles of the simulated populations. The pink region represents the range of the 50th percentile. The green line links the observed percentiles (10th, 50th and 90th) for each time point. Red regions represent where the observed data falls outside the ranges of the simulated percentiles.

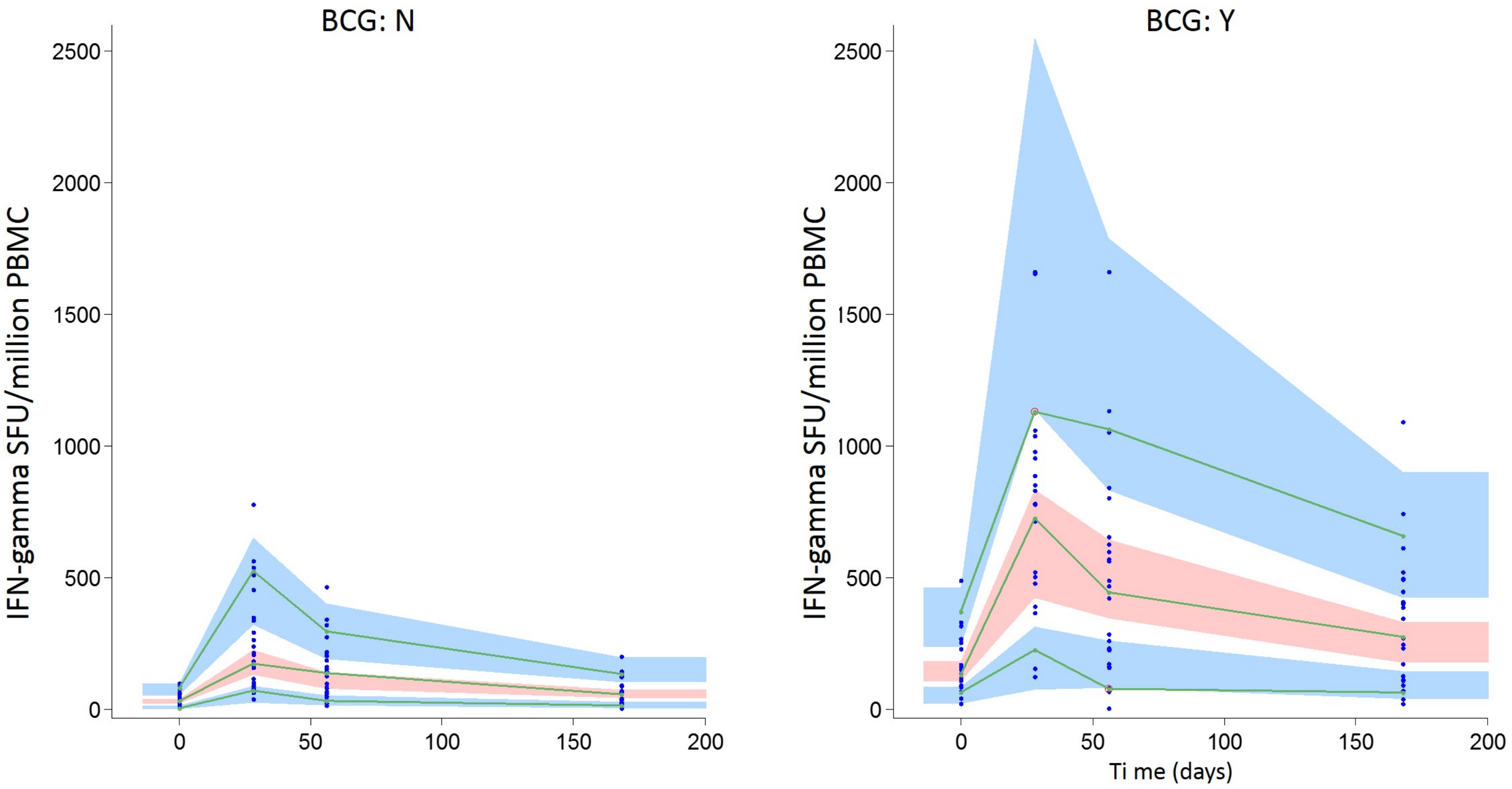

Figure S14: Visual predictive check plots for BCG: N and BCG: Y humans. Points represent the observed data. Blue regions represent the ranges of the 90th and 10th percentiles of the simulated populations. The pink region represents the range of the 50th percentile. The green line links the observed percentiles (10th, 50th and 90th) for each time point. Red regions represent where the observed data falls outside the ranges of the simulated percentiles.

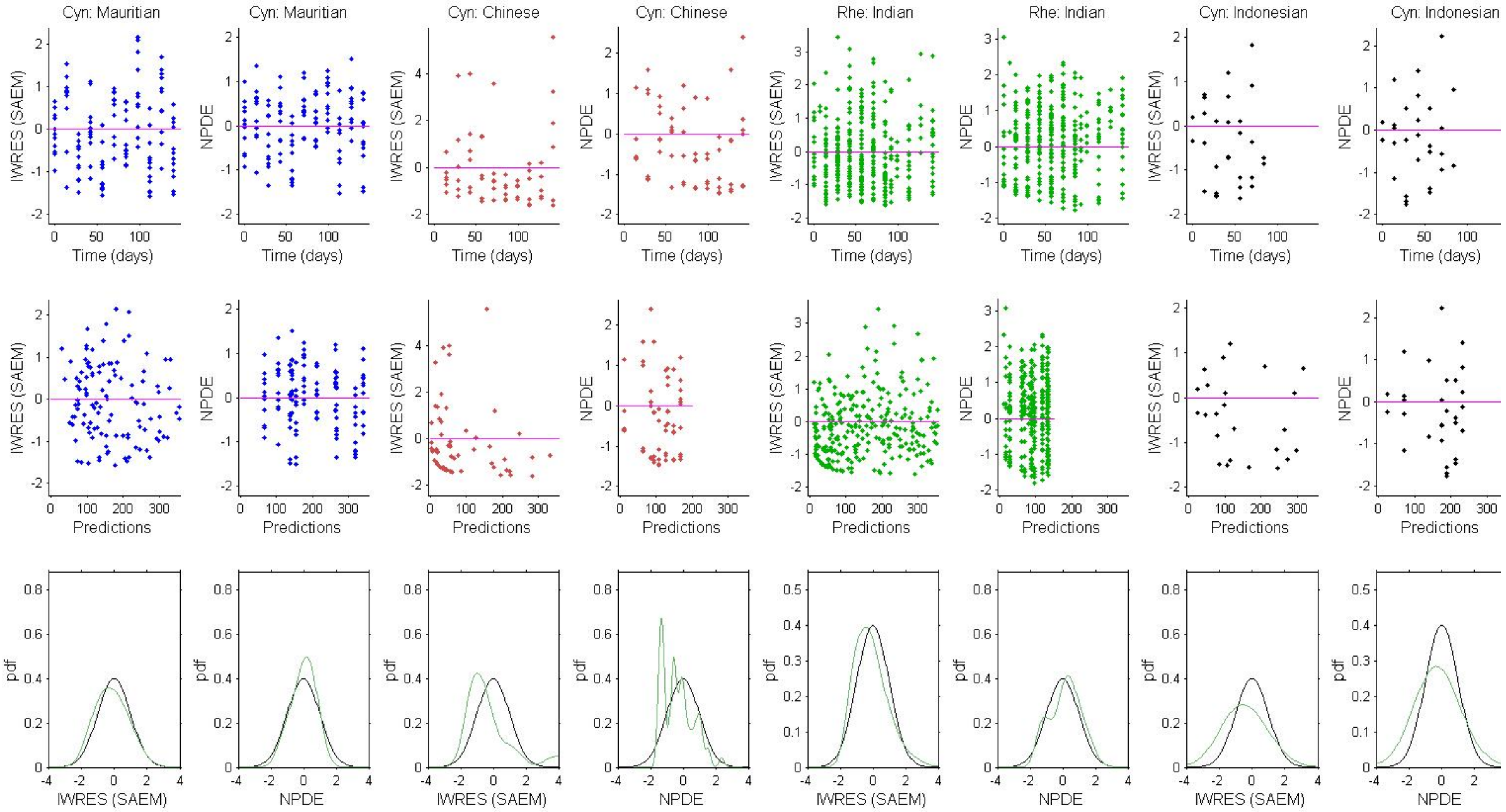

Figure S15: Residual (difference between data and total cells as predicted by the model) plots for macaque predicted total responses stratified by colony. The first row shows the individual weighted residuals (IWRES) and normalised prediction distribution errors (NPDE) using simulated individual parameters against time. The second row shows the residual error against the prediction. The bottom rows shows the distribution of the residuals compared to a Gaussian pdf curve so assess the normality of the residuals

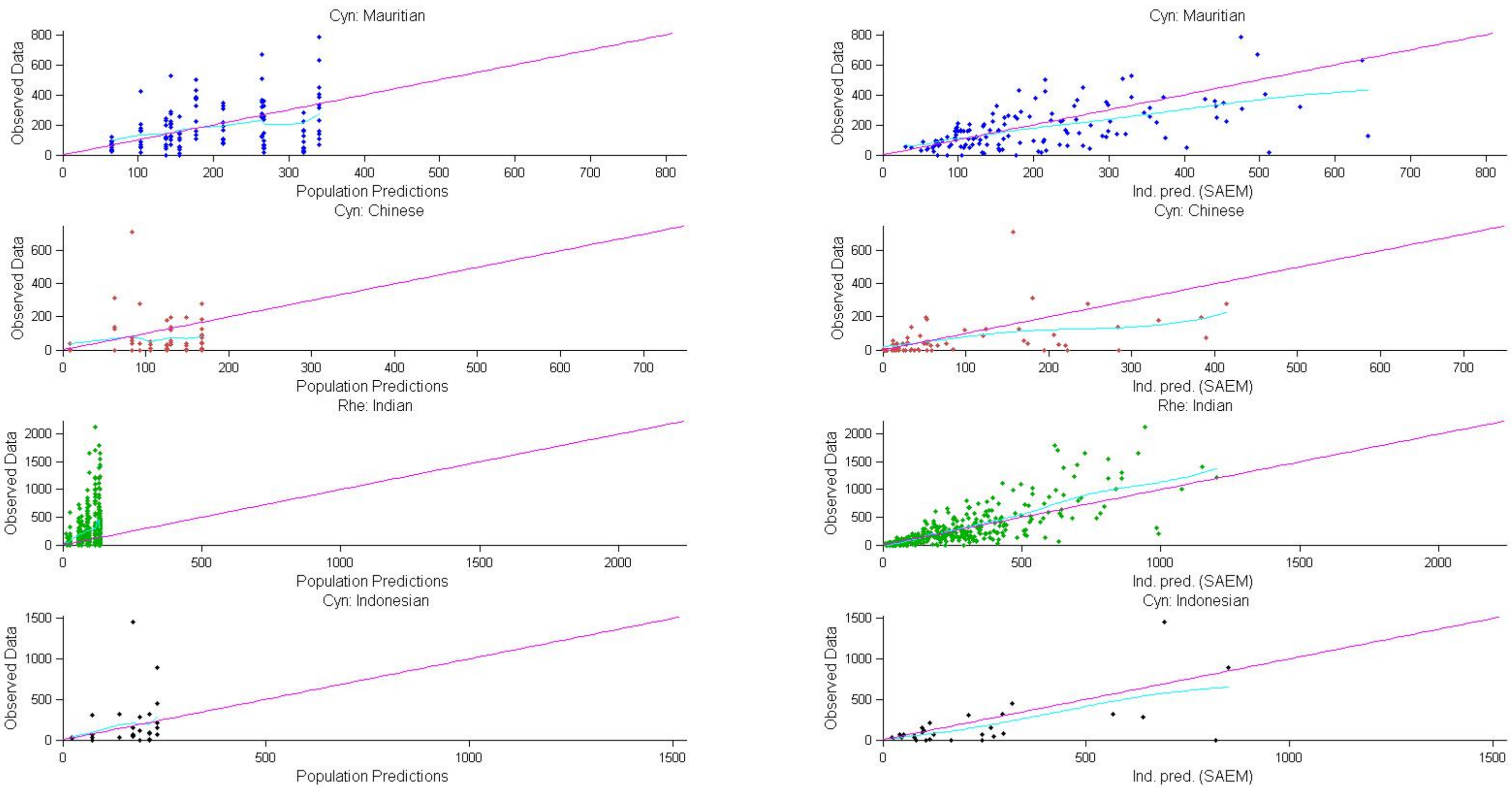

Figure S16: Macaque observed versus predicted IFN-γ total responses stratified by colony

Figures S15 and S16 show the accuracy of the model predictions for the macaque covariate model. Although the residuals are close to normally distributed (Figure S15), the Mauritian and Chinese cynomolgus macaque parameter sets over predict for higher IFN-γ responses (Figure S16). This could potentially be due to small colony populations. The Indian rhesus and Indonesian cynomolgus macaque predictions better describe the data. The VPC plots (Figure S13) show that, although the simulated percentile bands are wide for the Chinese, Indonesian and Mauritian cynomolgus macaques due to reduction in population size for these colonies, the empirical percentiles still fall (aside from variation between time points in the data) within the simulated bands.

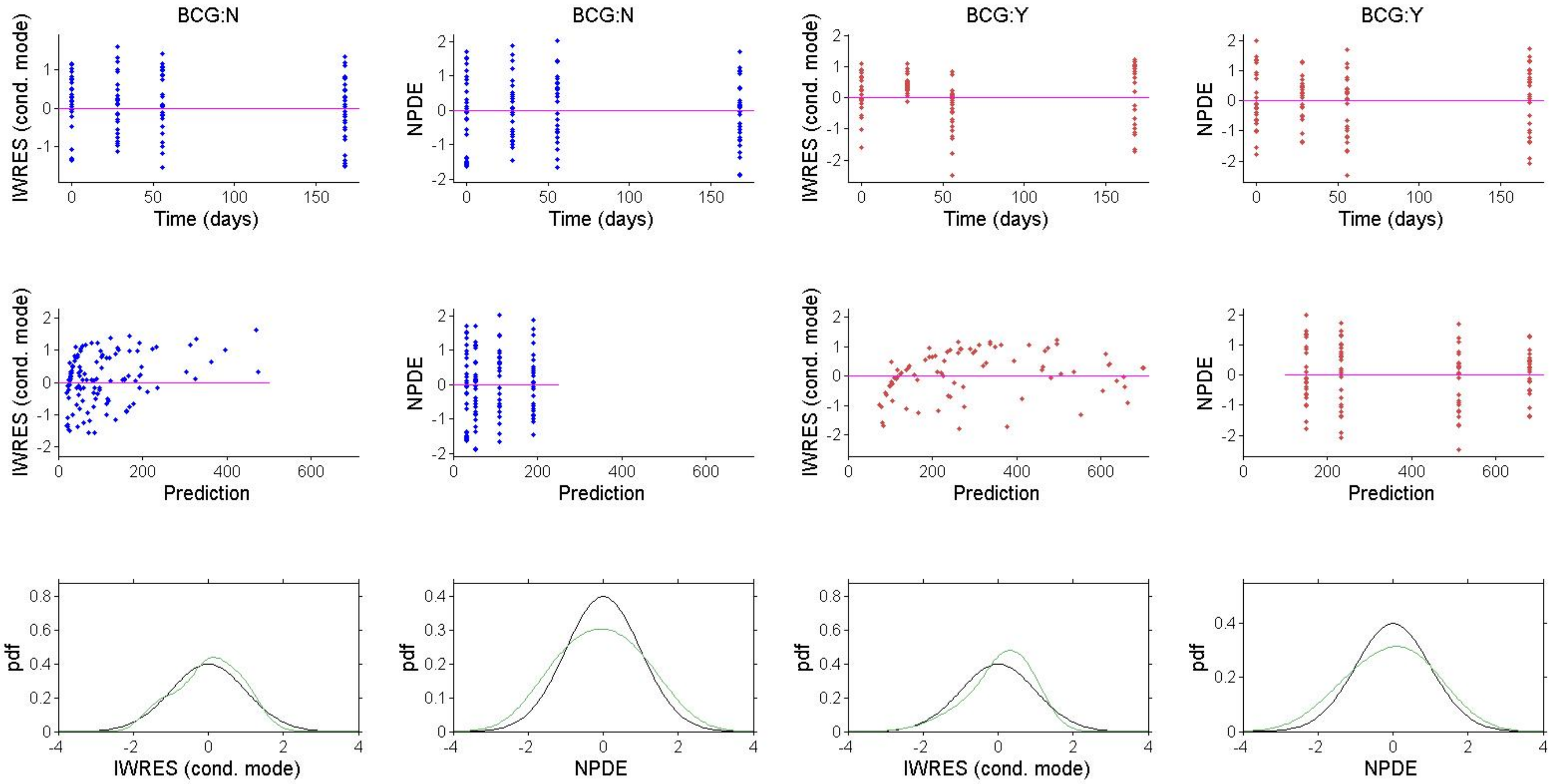

Figure S17: Residual (difference between data and total cells as predicted by the model) plots for human predicted total responses stratified by BCG status. The first row shows the individual weighted residuals (IWRES) and normalised prediction distribution errors (NPDE) using simulated individual parameters against time. The second row shows the residual error against the prediction. The bottom rows shows the distribution of the residuals compared to a Gaussian pdf curve so assess the normality of the residuals

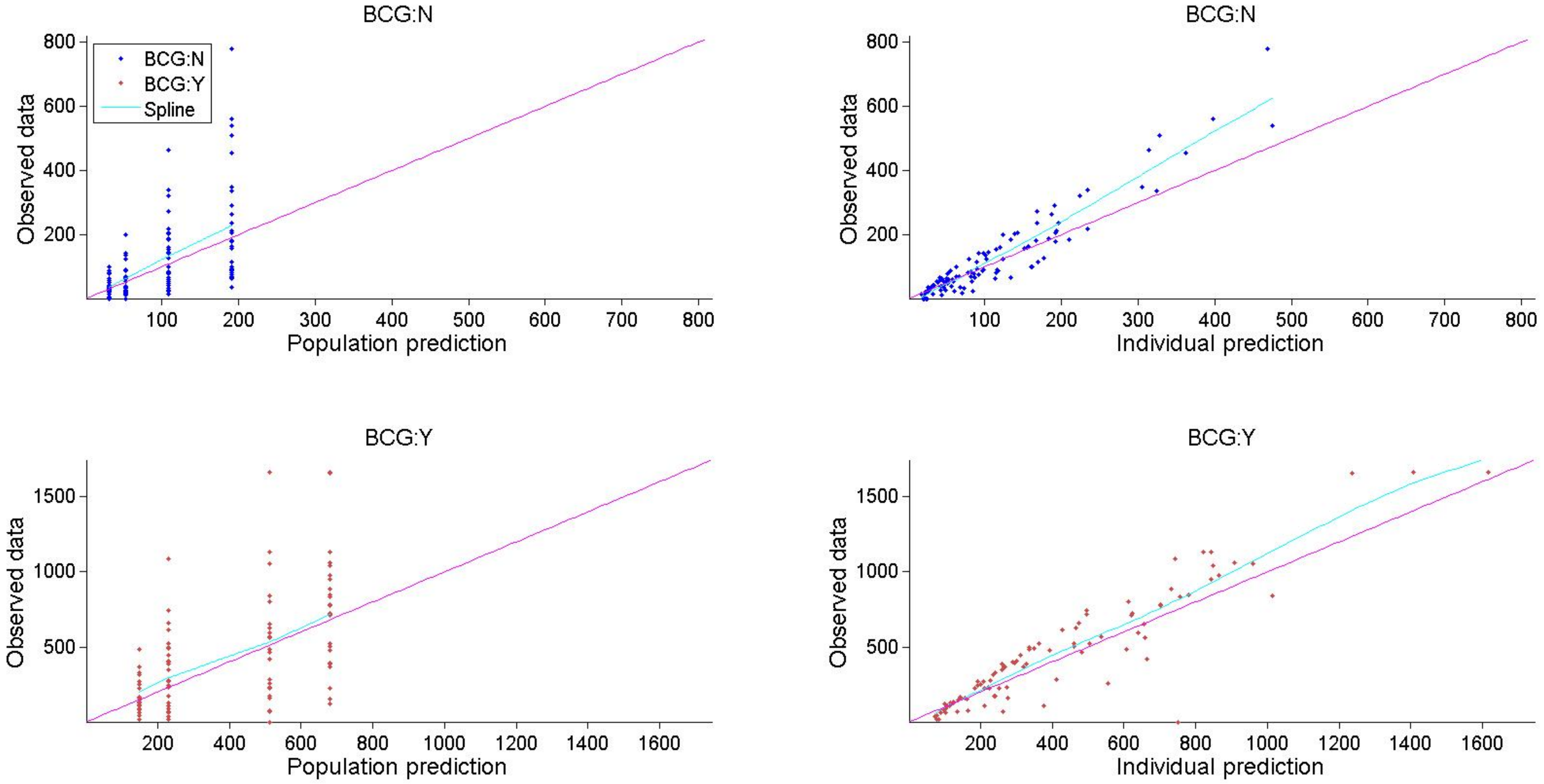

Figure S18: Human observed versus predicted IFN-γ responses stratified by BCG status

Figures S17 and S18 show the accuracy of the model predictions for the human covariate model. For the human subpopulations of BCG status, the residuals are close to normally distributed (Figure S17), however both BCG: Y and BCG: N parameter sets under predict the higher IFN-γ responses, more so for the BCG: N predictions (Figure S18). Despite this, the VPC plots (Figure S14) show that, the empirical percentiles still fall within the simulated bands.

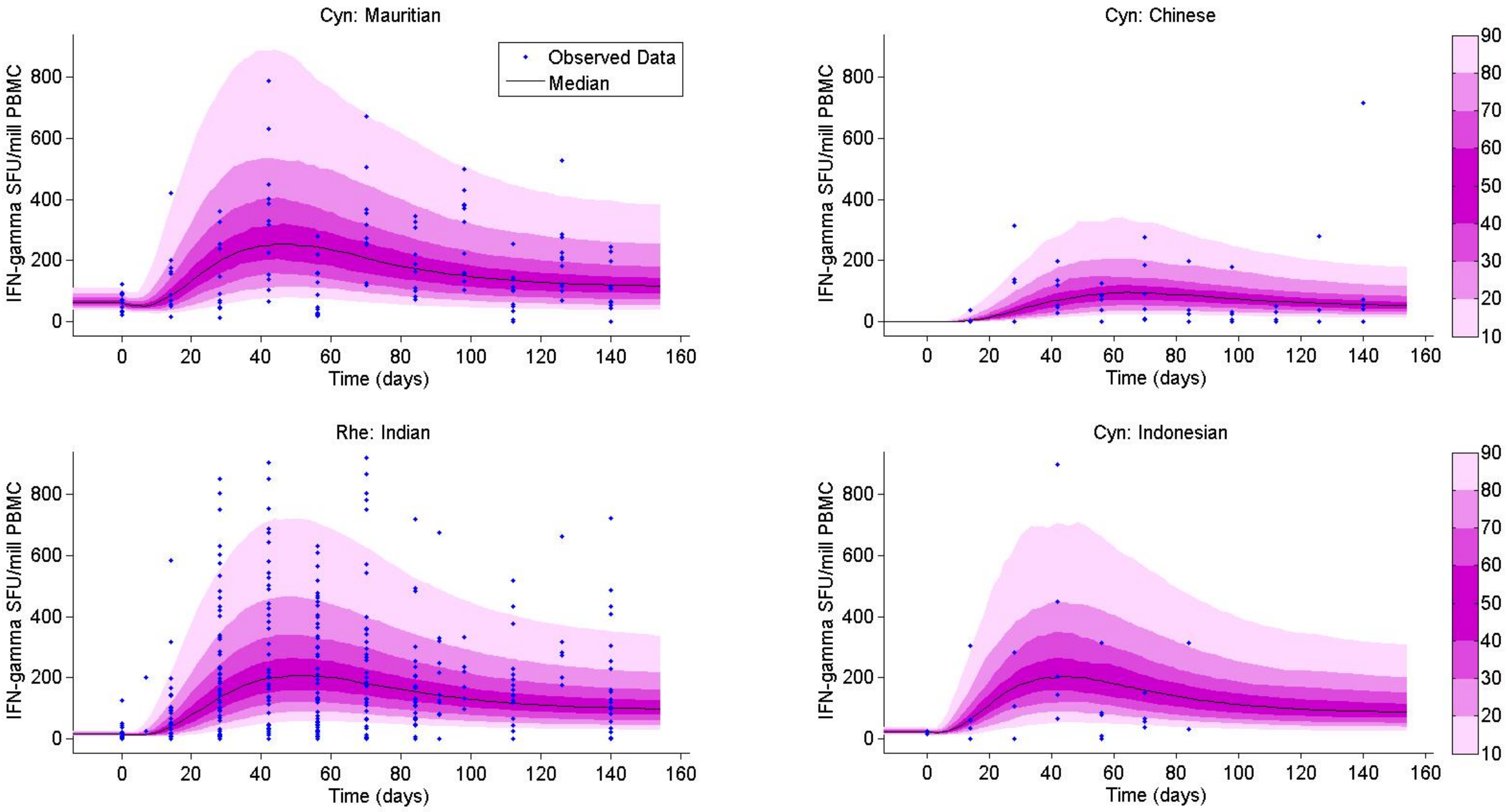

Figure S19: Prediction distribution plot for all colonies of macaque. Points represent the empirical data. The bands represent the 10th to 90th percentiles of the theoretical predictions using the predicted population parameters and associated variation for analysis 2 (Table 1). The black line shows the median total response prediction.

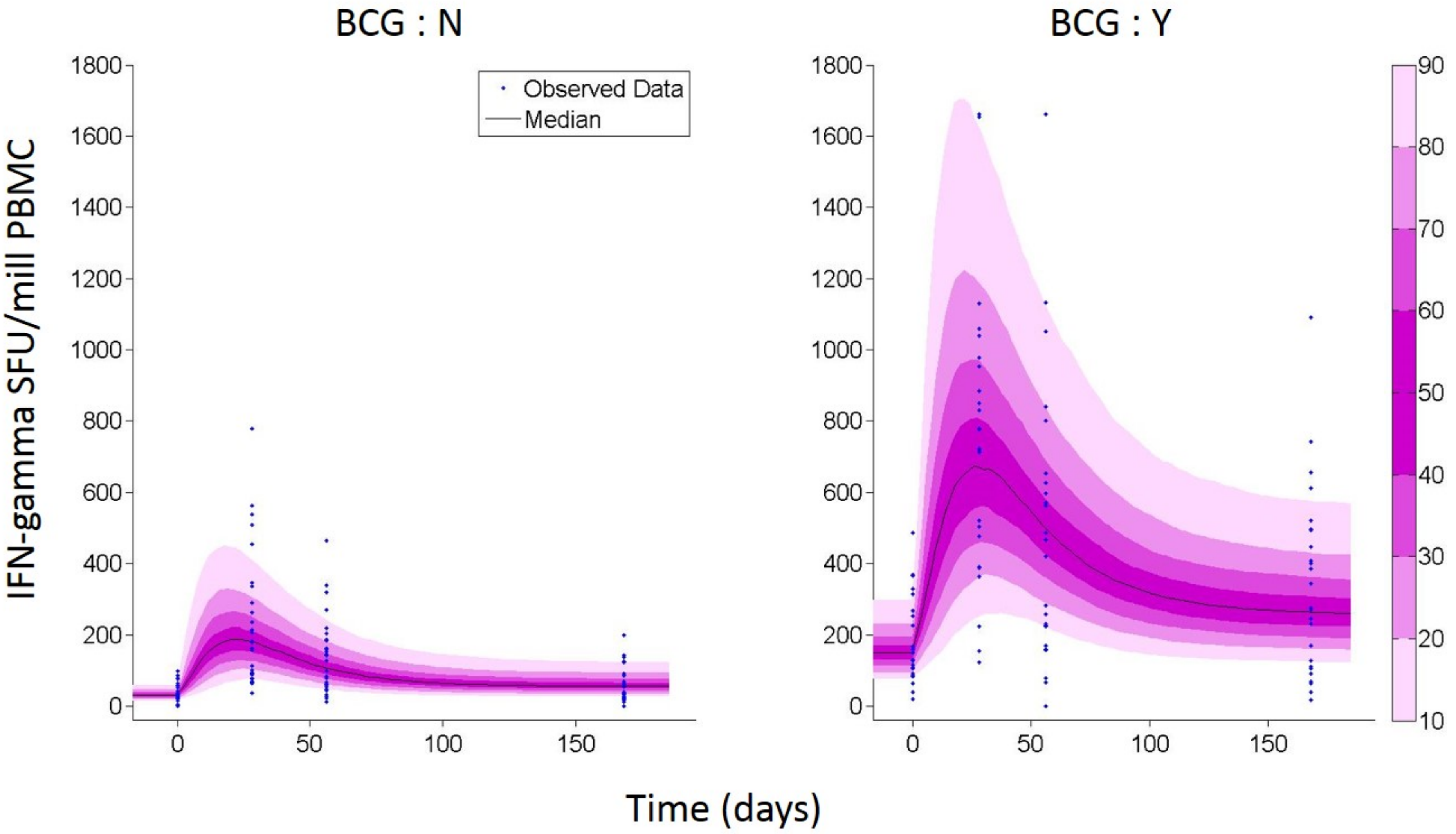

Figure S20: Prediction distribution plot for humans by BCG status subpopulation. Points represent the empirical data. The bands represent the 10th to 90th percentiles of the theoretical predictions using the predicted population parameters and associated variation for analysis 2 (Table 1). The black line shows the median total response prediction.

Analysis 3: Which macaque subpopulations best predicted immune responses in different human subpopulations?

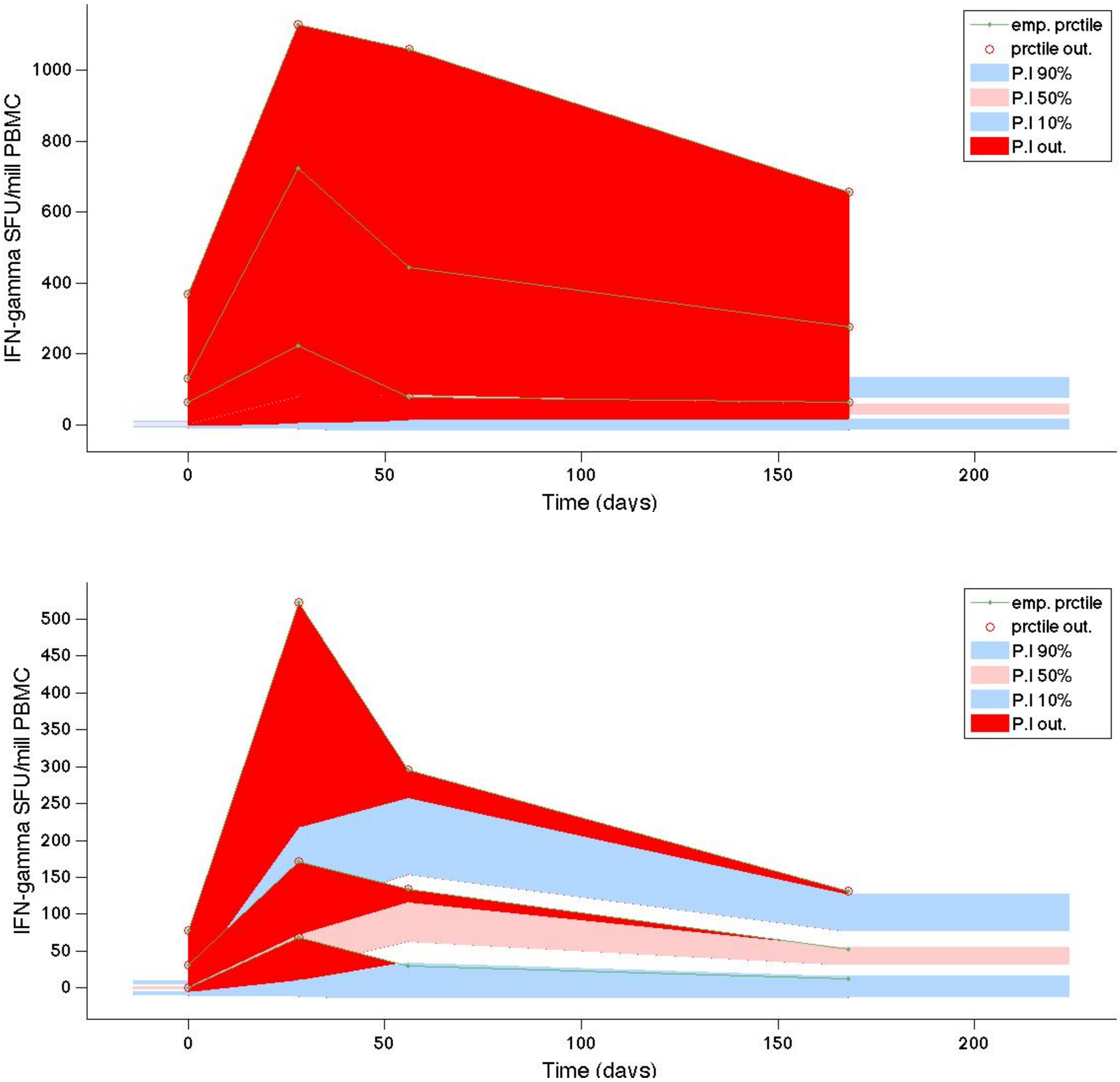

Figure S21: VPC plots for macaque estimated subpopulation-model parameters fit to the human BCG: Y data (top) and BCG: N data (bottom) for Chinese cynomolgus macaques. The green line links the observed percentiles (10th, 50th and 90th) for each time point. Blue regions represent the ranges of the 90th and 10th percentiles of the simulated populations time-matched to the observed data points. The pink region represents the range of the 50th percentile. Red regions represent where the observed data falls outside the ranges of the simulated percentiles.

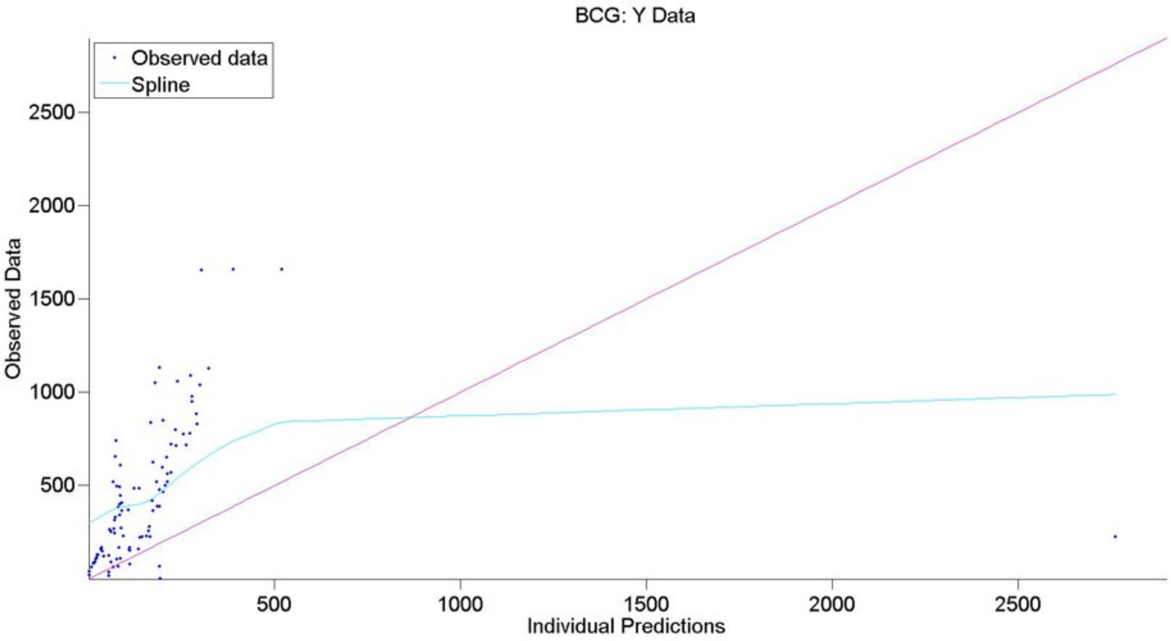

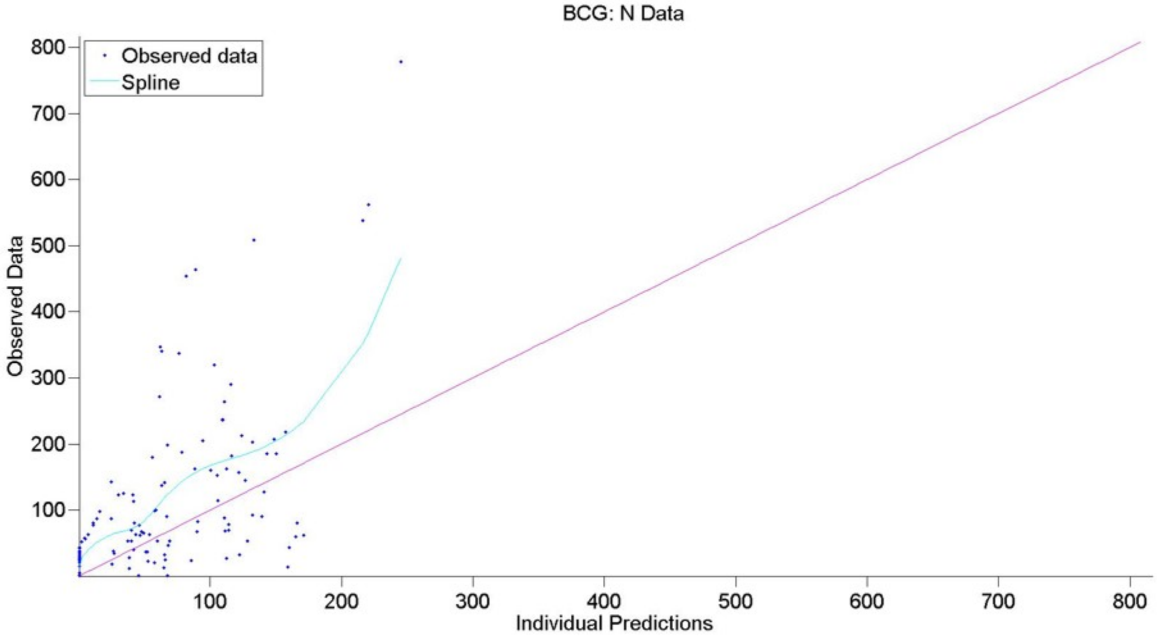

Figure S22: Individual empirical data versus individual prediction for macaque estimated subpopulation-model parameters fit to human BCG: Y data (top) and BCG: N data (bottom) for Chinese cynomolgus macaques.

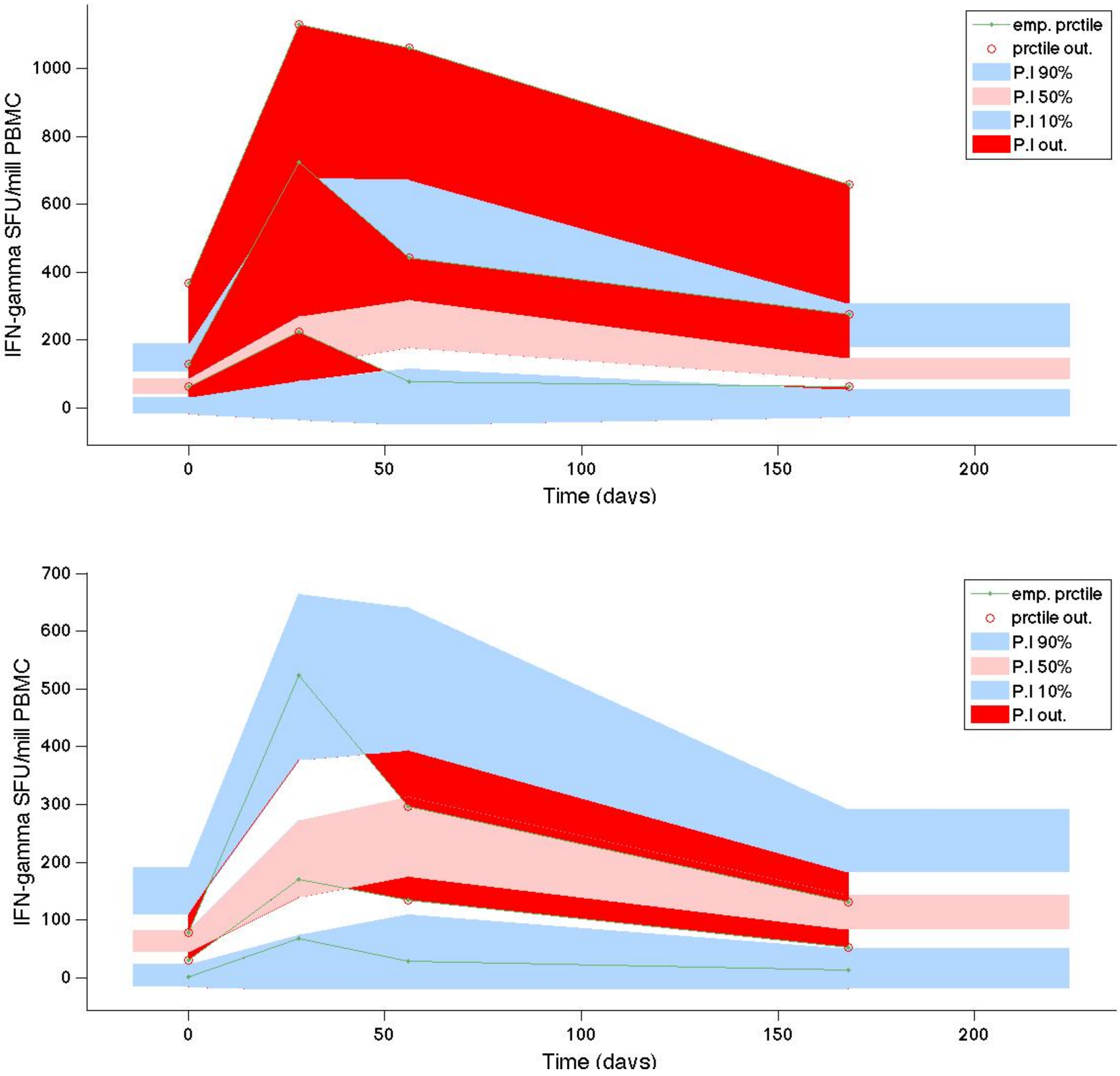

Figure S23: VPC plot for macaque estimated subpopulation-model parameters fit to the human BCG: Y data (top) and BCG: N data (bottom) for Mauritian cynomolgus macaques. The green line links the observed percentiles (10th, 50th and 90th) for each time point. Blue regions represent the ranges of the 90th and 10th percentiles of the simulated populations time-matched to the observed data points. The pink region represents the range of the 50th percentile. Red regions represent where the observed data falls outside the ranges of the simulated percentiles.

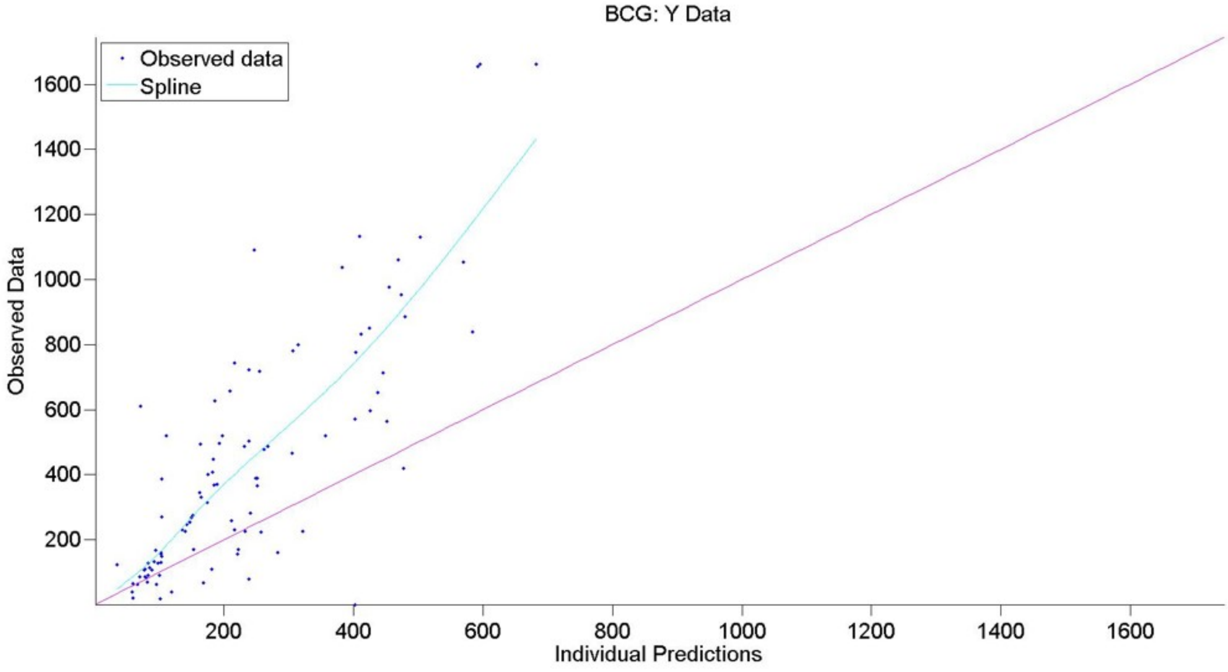

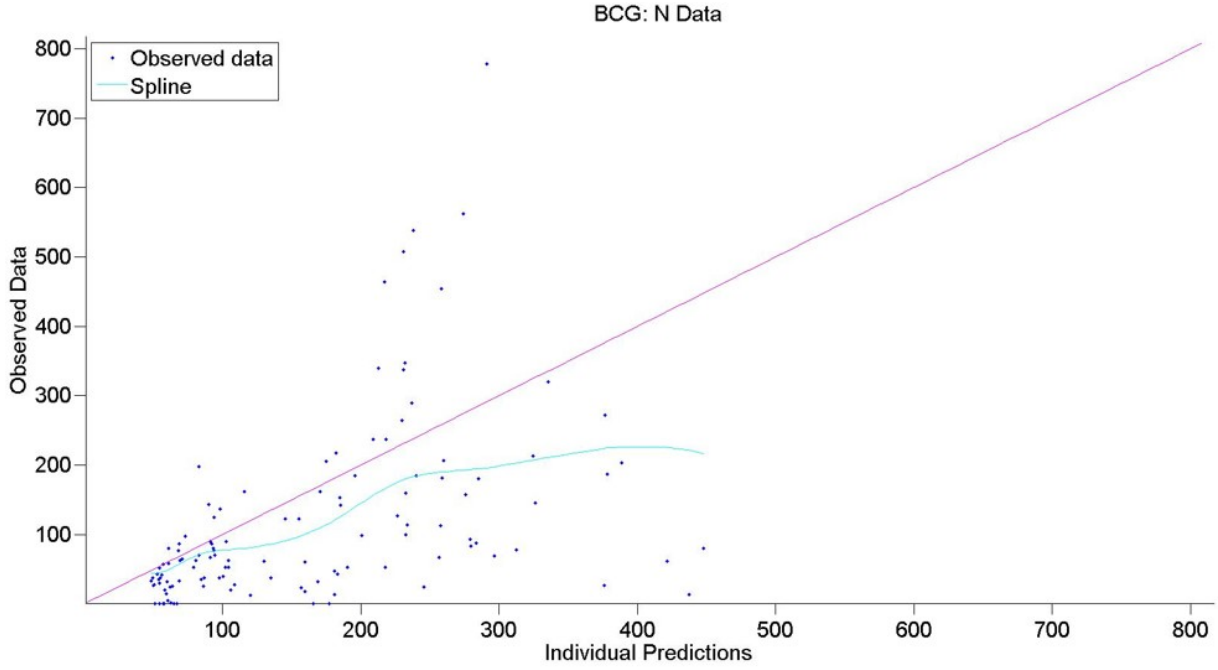

Figure S24: Individual empirical data versus individual prediction for macaque estimated subpopulation-model parameters fit to the human BCG: Y data (top) and BCG: N data (bottom) for Mauritian cynomolgus macaques.

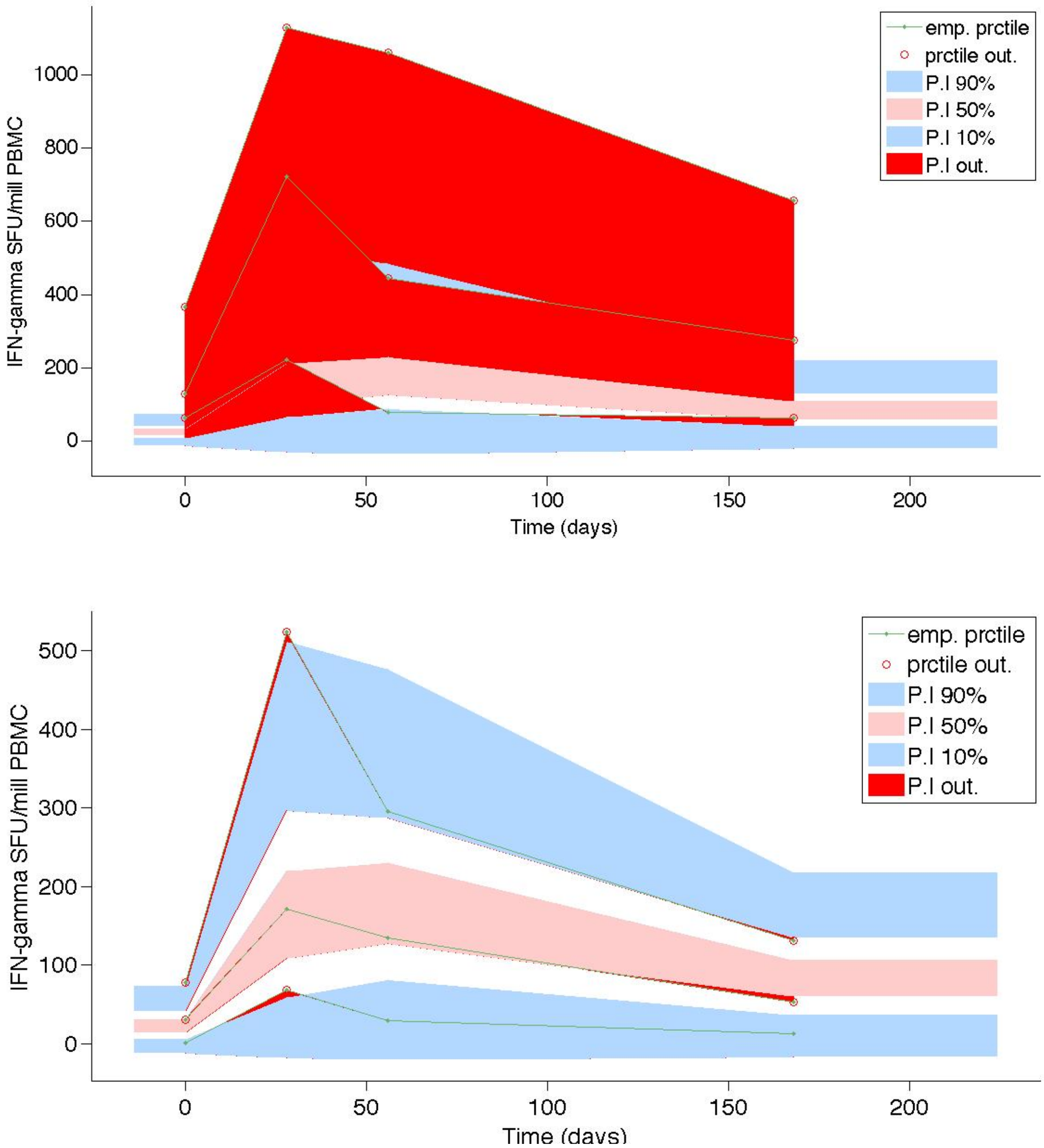

Figure S25: VPC plot for macaque estimated subpopulation-model parameters fit to the human BCG: Y data (top) and BCG: N data (bottom) for Indonesian cynomolgus macaques. The green line links the observed percentiles (10th, 50th and 90th) for each time point. Blue regions represent the ranges of the 90th and 10th percentiles of the simulated populations time-matched to the observed data points. The pink region represents the range of the 50th percentile. Red regions represent where the observed data falls outside the ranges of the simulated percentiles.

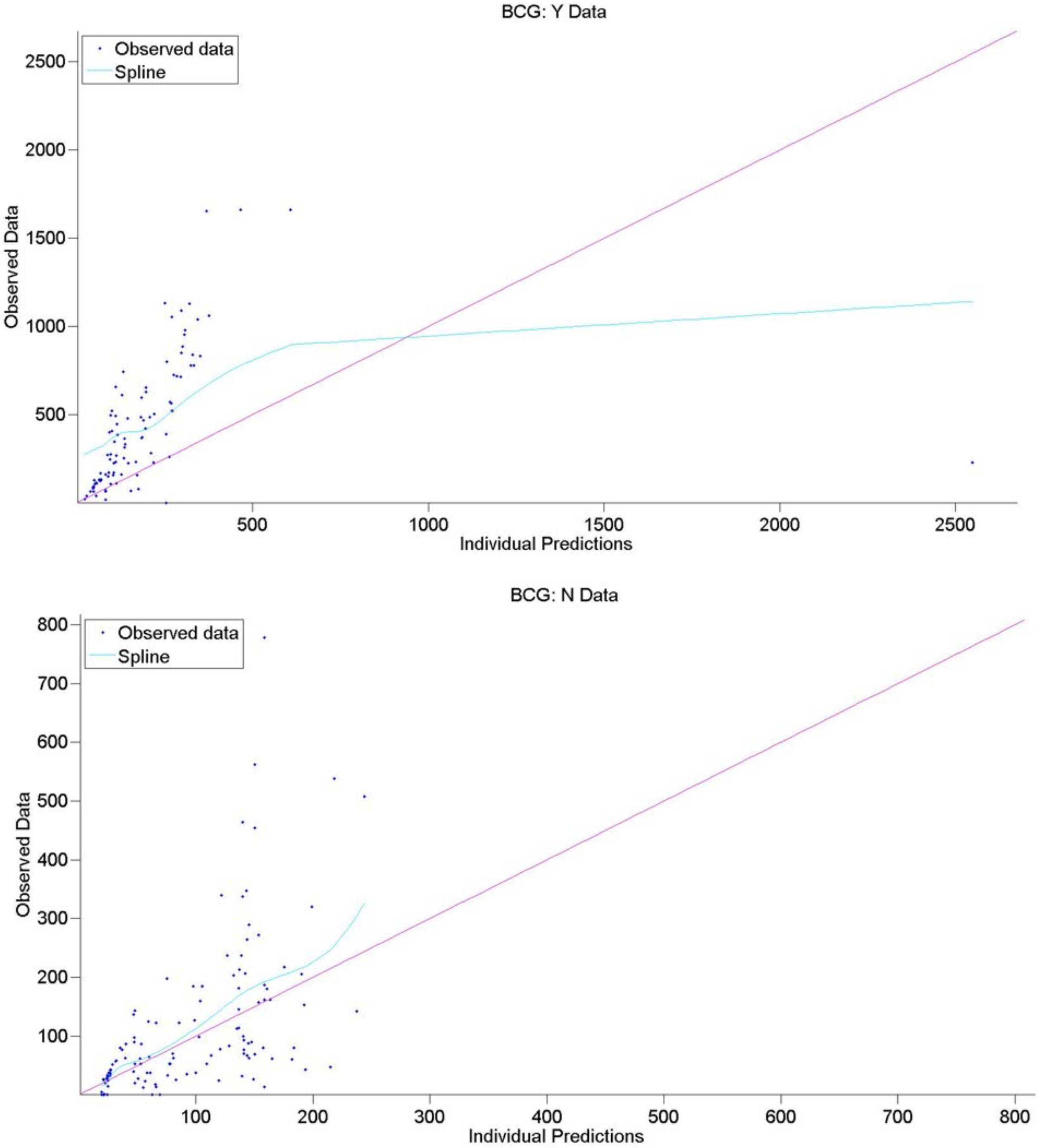

Figure S26: Individual empirical data versus individual prediction for macaque estimated subpopulation-model parameters fit to the human BCG: Y data (top) and BCG: N data (bottom) for Indonesian cynomolgus macaques.

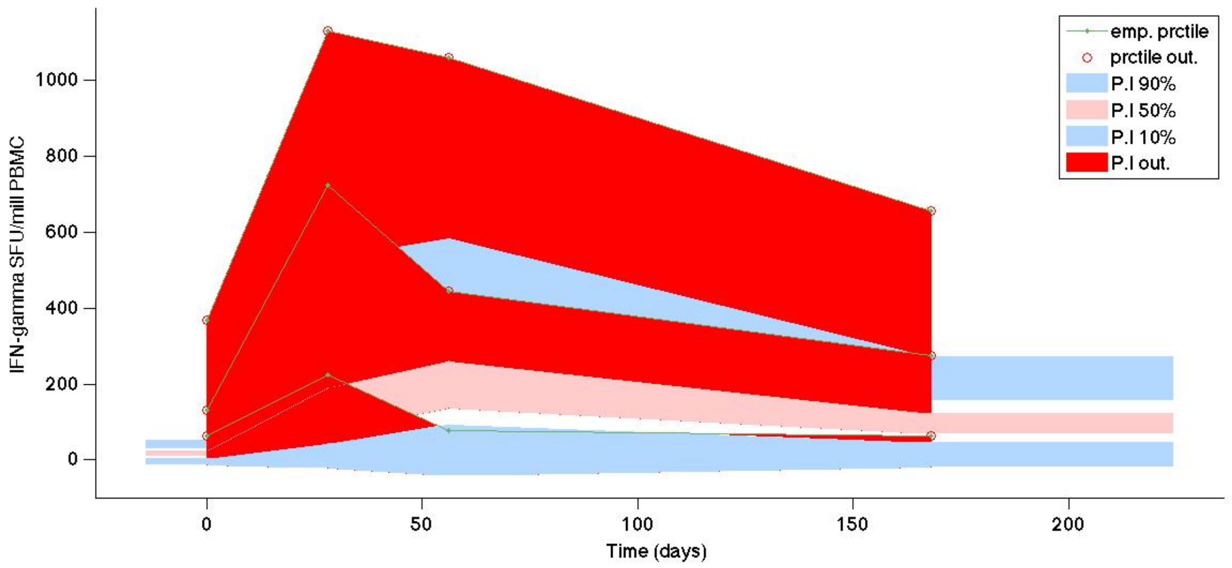

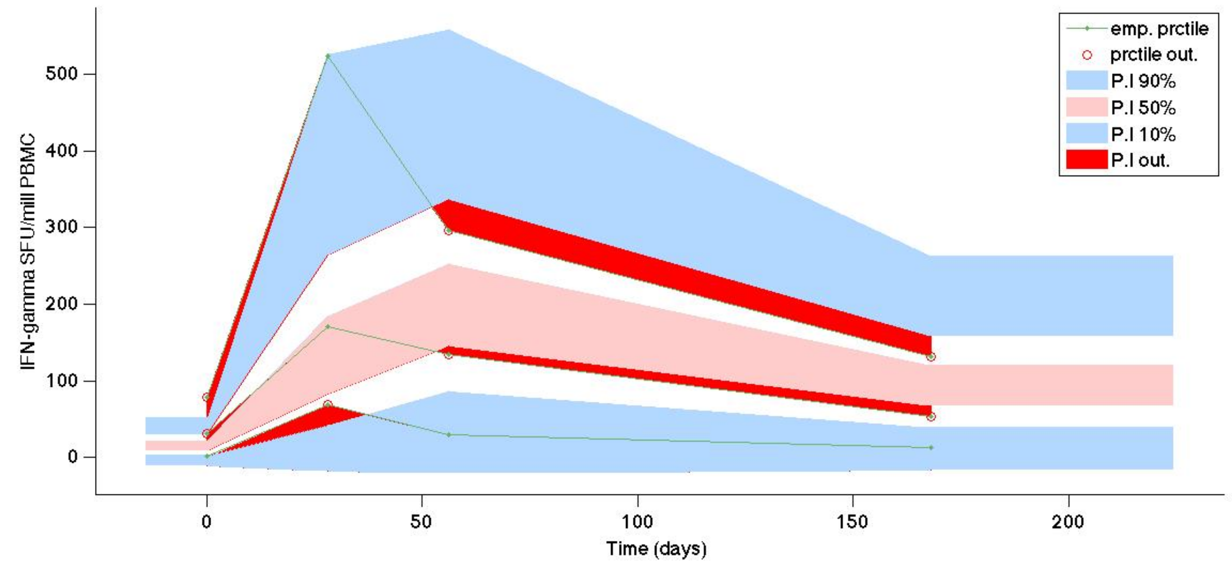

Figure S27: VPC plot for macaque estimated subpopulation-model parameters fit to the human BCG: Y data (top) and BCG: N data (bottom) for Indian rhesus macaques. The green line links the observed percentiles (10th, 50th and 90th) for each time point. Blue regions represent the ranges of the 90th and 10th percentiles of the simulated populations time-matched to the observed data points. The pink region represents the range of the 50th percentile. Red regions represent where the observed data falls outside the ranges of the simulated percentiles.

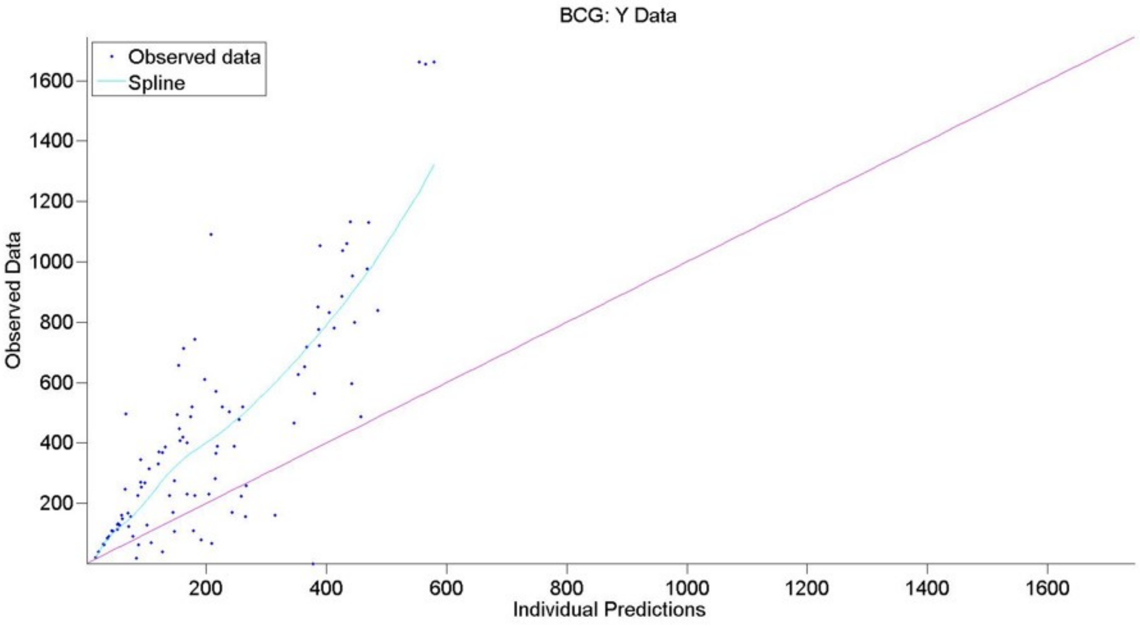

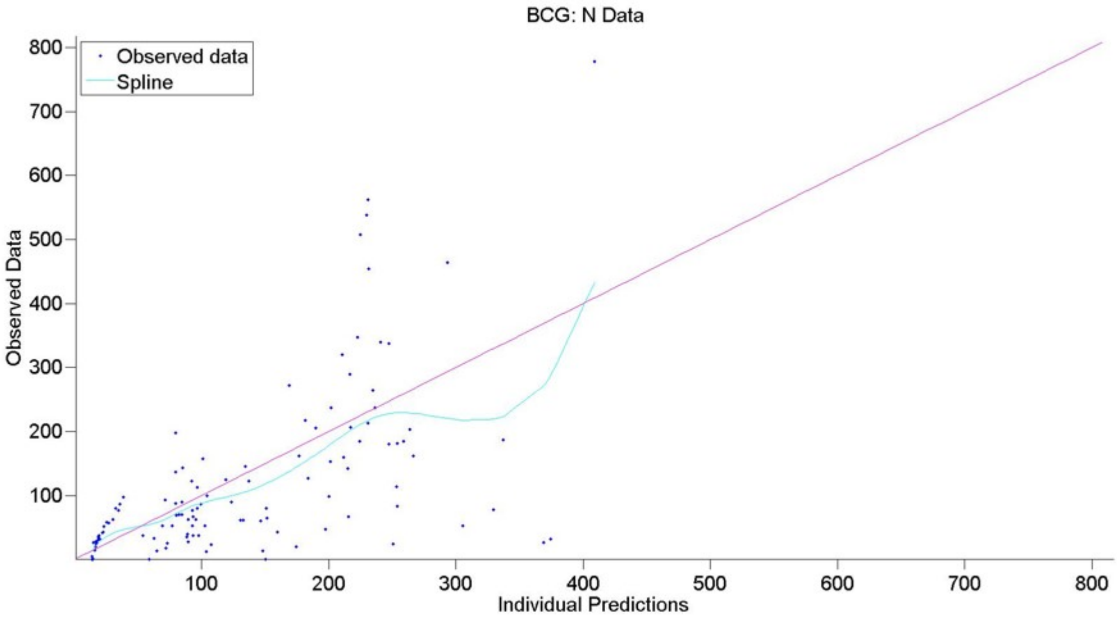

Figure S28: Individual empirical data versus individual prediction for macaque estimated subpopulation-model parameters fit to the human BCG: Y data (top) and BCG: N data (bottom) for Indian rhesus macaques.

3 Additional Discussion

Assumption	Implications for model
**<u>CD4 T cell stimulation greatly simplified</u>**<br>The immune response to vaccination is a complex network of cells and cytokines behaving nonlinearly over time. In the Th1 response to Mtb infection (or vaccination), innate and adaptive cells interact to optimise and maintain a protective response [7]. Very simply, cytokines secreted by innate cells after infection or vaccination, such as IL-12, work to stimulate adaptive cells to produce IFN-γ that both encourages innate cells to phagocytose bacteria and produce more IL-12 [8, 9]. As such, a feedback stimulation loop is established. In addition, to avoid an over-inflammatory response (which is harmful to the host) cytokines such as IL-10 are produced to regulate and dampen the immune response [10]. In the model, function δ is used to represent the delay of T cell initiation due to processes such as antigen processing and presentation and the decline of T cell responses due to depreciation of the required stimulation (creating a "n-shaped" curve). However, δ neglects the influence of stimulation amplification as a result of cytokine feedback loops, amongst other co-stimulation factors. As such, δ is a generalization of the complex networks required to protect against infection or vaccination and may not be as prolonged as required to generate a response to vaccination.	If data were available on IL-12 or other cytokines believed to be important to an immune response to BCG, It is possible that δ could be modelled as a parallel "innate response" compartmental model. Incorporating such a model would provide insight into the innate cell mechanisms and thus strengthen the conclusions we draw on the T cell dynamics.
**<u>Shape of stimulation curve, δ</u>**<br>The Gamma pdf distribution function fit well for δ for the BCG data in the analysis, so no other functional forms were tested. Although an abstract concept, it is possible that a different shape may be required if the model was to be applied to different type of vaccine (i.e. viral vector vaccines (e.g. novel TB vaccine MVA-85A) deliver a rapid "burst" of transitional effector cells compared to a live replicating vaccines (BCG) [communication, H. Fletcher]).	
**<u>No Terminal Effector cells</u>**<br>It is known that before TEM cells apoptose, they transition to a terminal effector phenotype [11]. We assume this process is incorporated into the μ_{TEM} parameter, i.e. for humans, the TEM cells will transition to terminal effector phenotype and apoptose on average after 12 days (Table 1 and Table S2). This assumption was made to simplify the model to avoid over-parameterisation.	To incorporate the terminal effector cells, a separate terminal effector compartment would be added where the proportion p of TEM cells would enter at a new rate β_{TEM} which would then be calibrated in Monolix. Terminal effector cells would then apoptose at a rate to be derived from literature.
**<u>No initial recruitment into resting memory compartment</u>**<br>The model assumes a linear progression from transitional effector memory to resting memory cell phenotype [12-14] . However a branched differentiation model, whereby memory cells progress directly from naive CD4 T cells and bypass the transitional effector stage, has been suggested [15, 16]. The determining factor as to which pathway is optimal is still not fully understood [17].	To incorporate a nonlinear effector-memory pathway into the model, a recruitment term like δ would be added to the memory compartment.
**<u>Transition and replication of transitional effector cells happens in Lymph node before entering the blood</u>**<br>The model assumes that the recruited transitional effector cells are former Mtb-specific naïve CD4 T cells that have clonally expanded within the *lymph node* and exited into the blood stream. Under this assumption, transitional effector cells do not replicate in this model. The rate of naïve CD4 T cell clonal expansion changes with time dependent on stimulation from innate processes and antigen presence [17] so could be considered to be incorporated into δ.	To incorporate replication of transitional effector cells into the model, a parameter R_E would be applied which would determine the rate at which replication occurs, dependent on the current transitional effector cell count.

Table S14: Main assumptions of the model and implications on challenging these assumptions

References

1. McShane, H., A.A. Pathan, C.R. Sander, S.M. Keating, S.C. Gilbert, K. Huygen, H.A. Fletcher, and A.V. Hill, *Recombinant modified vaccinia virus Ankara expressing antigen 85A boosts BCG-primed and naturally acquired antimycobacterial immunity in humans.* Nat Med, 2004. **10**(11): p. 1240-4.
2. Pathan, A.A., C.R. Sander, H.A. Fletcher, I. Poulton, N.C. Alder, N.E. Beveridge, K.T. Whelan, A.V. Hill, and H. McShane, *Boosting BCG with recombinant modified vaccinia ankara expressing antigen 85A: different boosting intervals and implications for efficacy trials.* PLoS One, 2007. **2**(10): p. e1052.
3. Fletcher, H.A., R. Tanner, R.S. Wallis, J. Meyer, Z.R. Manjaly, S. Harris, I. Satti, R.F. Silver, D. Hoft, B. Kampmann, K.B. Walker, H.M. Dockrell, U. Fruth, L. Barker, M.J. Brennan, and H. McShane, *Inhibition of mycobacterial growth in vitro following primary but not secondary vaccination with Mycobacterium bovis BCG.* Clin Vaccine Immunol, 2013. **20**(11): p. 1683-9.
4. Lavielle, M., *Mixed Effects Models for the Population Approach: Models, Tasks, Methods and Tools*. CRC Biostatistics series. 2015: Chapman & Hall. 365.
5. Raftery, A., *Bayesian Model Selection in Social Research.* Sociological Methodology, 1995. **25**: p. 111-163.
6. R, *R: A Language and Environment*, D.C. Team, Editor. 2005, R Foundation for Statistical Computing (http://www.r-project.org/): Vienna, Austria.
7. O'Garra, A., P.S. Redford, F.W. McNab, C.I. Bloom, R.J. Wilkinson, and M.P. Berry, *The immune response in tuberculosis.* Annu Rev Immunol, 2013. **31**: p. 475-527.
8. Jouanguy, E., R. Doffinger, S. Dupuis, A. Pallier, F. Altare, and J.L. Casanova, *IL-12 and IFN-gamma in host defense against mycobacteria and salmonella in mice and men.* Curr Opin Immunol, 1999. **11**(3): p. 346-51.
9. Flynn, J.L., *Immunology of tuberculosis and implications in vaccine development.* Tuberculosis (Edinb), 2004. **84**(1-2): p. 93-101.
10. Mayer-Barber, K.D., B.B. Andrade, S.D. Oland, E.P. Amaral, D.L. Barber, J. Gonzales, S.C. Derrick, R. Shi, N.P. Kumar, W. Wei, X. Yuan, G. Zhang, Y. Cai, S. Babu, M. Catalfamo, A.M. Salazar, L.E. Via, C.E. Barry, 3rd, and A. Sher, *Host-directed therapy of tuberculosis based on interleukin-1 and type I interferon crosstalk.* Nature, 2014. **511**(7507): p. 99-103.
11. Seder, R.A., P.A. Darrah, and M. Roederer, *T-cell quality in memory and protection: implications for vaccine design.* Nat Rev Immunol, 2008. **8**(4): p. 247-58.
12. Jacob, J. and D. Baltimore, *Modelling T-cell memory by genetic marking of memory T cells in vivo.* Nature, 1999. **399**(6736): p. 593-7.
13. Opferman, J.T., B.T. Ober, and P.G. Ashton-Rickardt, *Linear differentiation of cytotoxic effectors into memory T lymphocytes.* Science, 1999. **283**(5408): p. 1745-8.
14. Hu, H., G. Huston, D. Duso, N. Lepak, E. Roman, and S.L. Swain, *CD4(+) T cell effectors can become memory cells with high efficiency and without further division.* Nat Immunol, 2001. **2**(8): p. 705-10.
15. Laouar, A., M. Manocha, V. Haridas, and N. Manjunath, *Concurrent generation of effector and central memory CD8 T cells during vaccinia virus infection.* PLoS One, 2008. **3**(12): p. e4089.
16. Iezzi, G., D. Scheidegger, and A. Lanzavecchia, *Migration and function of antigen-primed nonpolarized T lymphocytes in vivo.* J Exp Med, 2001. **193**(8): p. 987-93.
17. Abbas, A., A. Lichtman, and S. Pillai, *Cellular and Molecular Immunology*. 8 ed. 2015: Elsevier Saunders.
